# Single-nucleus transcriptomic profiling of human orbitofrontal cortex reveals convergent effects of aging and psychiatric disease

**DOI:** 10.1038/s41593-024-01742-z

**Published:** 2024-09-03

**Authors:** Anna S. Fröhlich, Nathalie Gerstner, Miriam Gagliardi, Maik Ködel, Natan Yusupov, Natalie Matosin, Darina Czamara, Susann Sauer, Simone Roeh, Vanessa Murek, Chris Chatzinakos, Nikolaos P. Daskalakis, Janine Knauer-Arloth, Michael J. Ziller, Elisabeth B. Binder

**Affiliations:** 1 Department of Genes and Environment, Max Planck Institute of Psychiatry, Munich, Germany; 2 International Max Planck Research School for Translational Psychiatry, Munich, Germany; 3 Institute of Computational Biology, Helmholtz Zentrum München, Neuherberg, Germany; 4 Department of Psychiatry, University of Münster, Münster, Germany; 5 School of Medical Sciences, Faculty of Medicine and Health, University of Sydney, Camperdown, New South Wales Australia; 6 Charles Perkins Centre, University of Sydney, Camperdown, New South Wales Australia; 7 Department of Psychiatry, McLean Hospital, Harvard Medical School, Belmont, MA USA; 8 Stanley Center for Psychiatric Research, Broad Institute of MIT and Harvard, Cambridge, MA USA; 9 Department of Psychiatry and Behavioral Sciences, Institute for Genomics in Health, SUNY Downstate Health Sciences University, Brooklyn, NY USA; 10 Department of Psychiatry and Behavioral Sciences, Emory University School of Medicine, Atlanta, GA USA

**Keywords:** Neural ageing, Transcriptomics, Cellular neuroscience, Psychiatric disorders

## Abstract

Aging is a complex biological process and represents the largest risk factor for neurodegenerative disorders. The risk for neurodegenerative disorders is also increased in individuals with psychiatric disorders. Here, we characterized age-related transcriptomic changes in the brain by profiling ~800,000 nuclei from the orbitofrontal cortex from 87 individuals with and without psychiatric diagnoses and replicated findings in an independent cohort with 32 individuals. Aging affects all cell types, with *LAMP5*^+^*LHX6*^+^ interneurons, a cell-type abundant in primates, by far the most affected. Disrupted synaptic transmission emerged as a convergently affected pathway in aged tissue. Age-related transcriptomic changes overlapped with changes observed in Alzheimer’s disease across multiple cell types. We find evidence for accelerated transcriptomic aging in individuals with psychiatric disorders and demonstrate a converging signature of aging and psychopathology across multiple cell types. Our findings shed light on cell-type-specific effects and biological pathways underlying age-related changes and their convergence with effects driven by psychiatric diagnosis.

## Abstract (web summary)

Single-cell profiling in the human cortex reveals aging-associated transcriptomic changes across all brain cell types, which overlap with effects with Alzheimer’s disease and show a convergent signature with psychopathology across multiple cell types.

## Main

Aging is a complex, not yet fully understood biological process, where changes at the level of molecules, cells and organs lead to alterations in function and physiology. The aging brain is characterized by structural and functional remodeling, especially in the prefrontal cortex and white matter tracts, ultimately affecting cognition and memory^1^. At the cellular level, reduction in spine density, axonal transport and synapse number, changes in neurotransmitter levels and mitochondrial dysfunction and oxidative damage have been described in the aging brain^2^.

Age represents the strongest risk factor for neurodegenerative disorders, suggesting that certain age-related changes could be directly involved in disease etiology. Given the increasing life expectancy of current societies, accompanied by a rise in the prevalence of neurodegenerative disorders, it is of great importance to better characterize underlying mechanisms of normal and pathological aging. Moreover, studies indicate common biological pathways affected by aging and psychiatric disorders^3^, another disease group with increasing prevalence and substantial socioeconomic burden. Transcriptomic and neuroimaging studies suggest that psychiatric disorders, such as schizophrenia (SCZ), are associated with accelerated brain age^4,5^.

Our current knowledge of the transcriptomic changes involved in brain aging is mainly limited to studies in other species, such as mice^6^ and nonhuman primates^7^, and to bulk human postmortem tissue^8,9^. Some studies^10,11^ in model organisms have implemented single-cell RNA sequencing to decipher cell-type-specific age-associated changes in gene expression. In humans, a recent single-nucleus RNA-sequencing (snRNA-seq) study^12^ examined changes in gene expression latent factors with aging, including in the context of SCZ. Understanding the unique transcriptomic effects of age for specific genes in individual cell types in the human brain and mapping shared or divergent alterations and affected molecular pathways and which cell types are most affected are important steps toward the development of potential therapeutic interventions to prevent or treat age-associated pathologies.

In this study, we profiled single-nucleus transcriptomes of the orbitofrontal cortex (OFC) of a cohort of 87 individuals ranging from 26 to 84 years of age. We focused on the OFC as it has an important role in cognitive functions^13^, suffers structural and functional decline during aging^14^ and is implicated in the pathophysiology of neuropsychiatric diseases^15,16^. The cohort contained neurotypical individuals and individuals diagnosed with a psychiatric disorder, mainly SCZ. With ~800,000 nuclei profiled, we provide a comprehensive dataset of age-associated genes, pathways and affected cell types that allowed us to analyze possible convergence with neurodegenerative and psychiatric diseases.

## Results

### Single-nucleus profiling of the human OFC

**Fig. 1:**
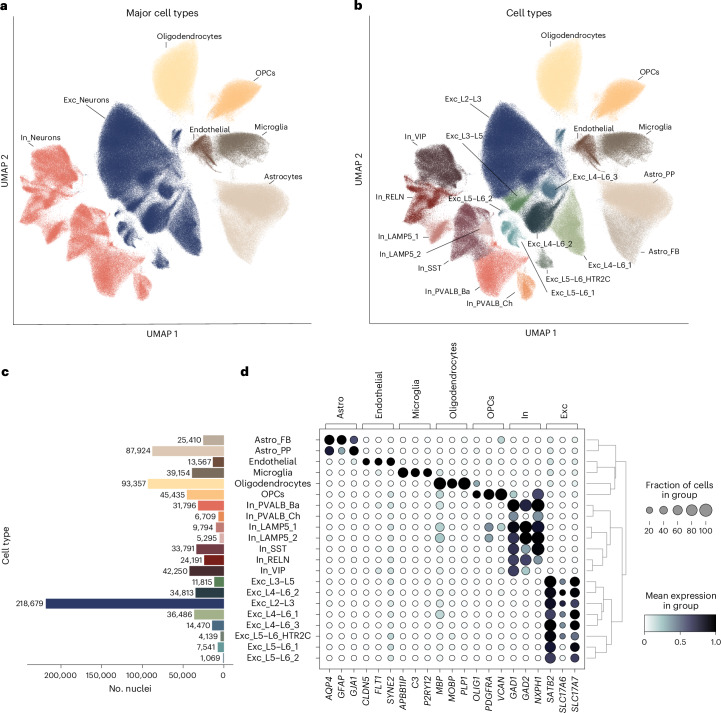
Identification of cell types. **a**,**b**, Uniform manifold approximation and projection (UMAP) showing ~800,000 nuclei from the OFC from 87 donors colored by major cell-type cluster (**a**) and individual cell-type cluster (**b**). Cell-type annotation was performed using a label transfer algorithm, followed by manual curation based on marker genes described in the literature. **c**, Bar plot depicting the number of nuclei per individual cell-type cluster. **d**, Left, dot plot showing the expression of representative marker genes, which are grouped by major cell types. The size of the dot represents the percentage of nuclei expressing the gene, and the color indicates the mean expression level. Right, dendrogram showing the relationship between identified cell-type clusters based on similarity in gene expression; Astro_FB, fibrous astrocytes; Astro_PP, protoplasmic astrocytes; Exc, excitatory; In, inhibitory; L, cortical layer; Ba, basket; Ch, chandelier; PVALB, parvalbumin.

**Extended Data Fig. 1:**
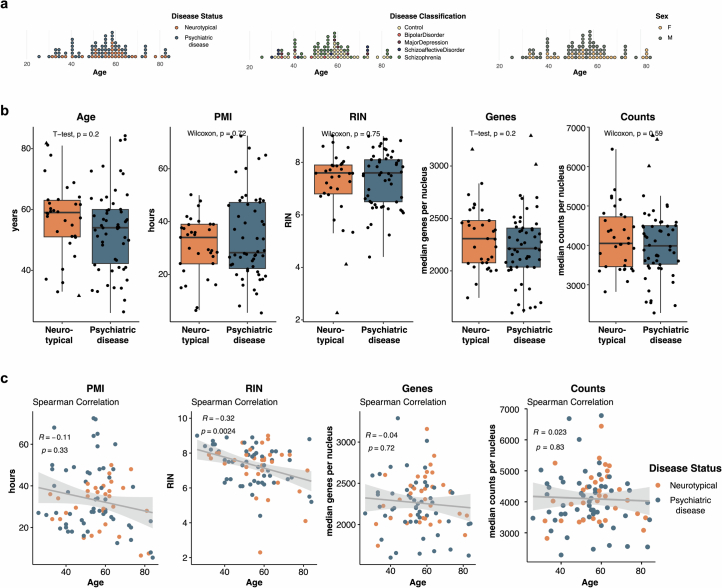
Cohort Parameters. **a**, Age distribution of cohort colored by controls and cases (neurotypical individuals and individuals with a pyschiatric disease) (left), colored by disease classification (middle), and colored by females (F) and males (M) (right) used for the single-nucleus RNA sequencing. **b**, Boxplots showing (from left to right) age, post-mortem interval (PMI), RNA integrity number (RIN), median number of genes and median number of counts between individuals with a pyschiatric disease (N = 54) and neurotypical individuals (N = 33). The type of test (as indicated in the plots) used was dependent on whether the data was normally distributed (t-test) or not (Mann-Whitney U test); tests were two-sided. Boxplots show the median (center), IQR (bounds of box), and whiskers extending to either the maxima/minima or to the median ± 1.5× IQR, whichever is nearest. Triangles indicate outliers. **c**, Spearman correlation (two-sided) of age and (from left to right) PMI, RIN, median number of genes and median number of counts.

**Extended Data Fig. 2:**
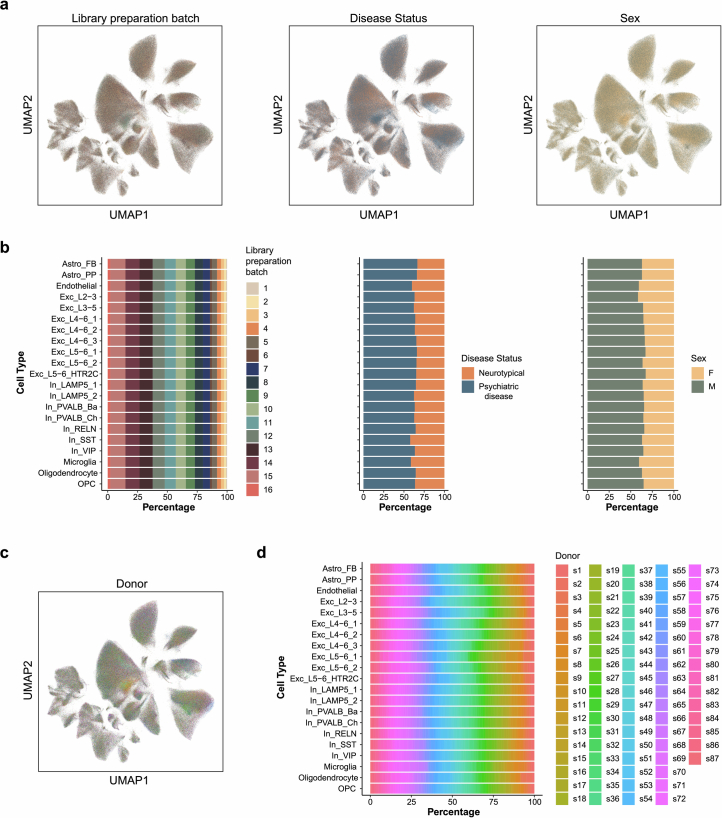
Clustering and experimental variables. **a**, Uniform Manifold Approximation and Projection (UMAP) showing ~800 000 nuclei from 87 donors from the orbitofrontal cortex colored by (from left to right) experimental batch, disease status, and sex indicating that clusters were not driven by these parameters. **b**, Stacked barplots showing the percentage contribution to the respective cell type cluster (from left to right) from experimental batch, disease status and sex. **c**, UMAP showing ~800 000 nuclei from 87 donors from the orbitofrontal cortex colored by donor. **d**, Stacked barplot showing the percentage contribution to the respective cell type cluster from each donor.

**Extended Data Fig. 3:**
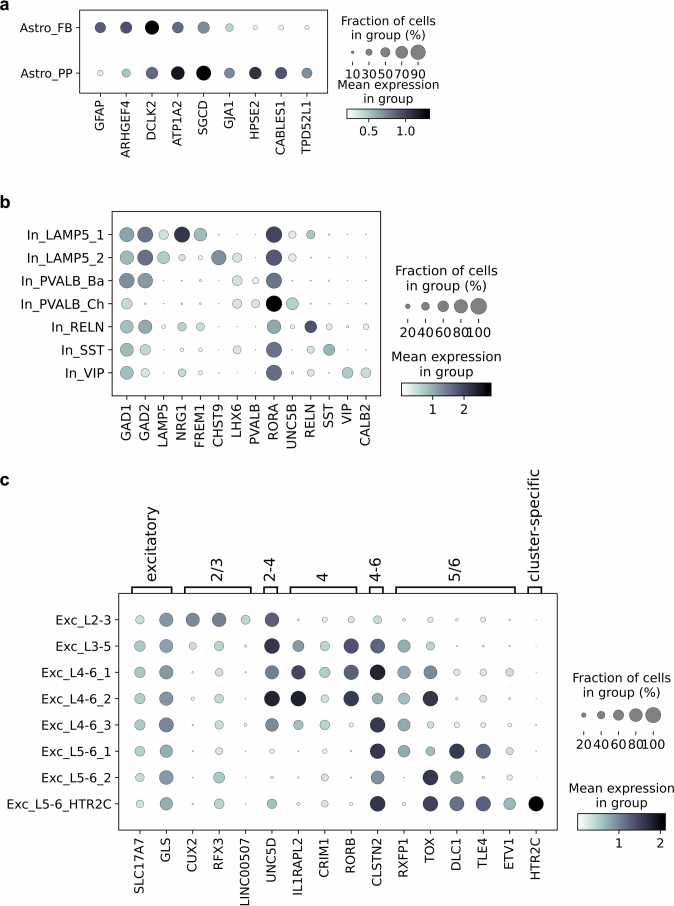
Cell type makers. **a-c**, Dotplot showing the expression of representative marker genes of cell subtypes for astrocyte subtypes (**a**), inhibitory neuron subtypes (**b**) and excitatory neuron subtypes (**c**). The size of the dot represents the fraction (%) of nuclei expressing the gene and the color indicates the mean expression level.

To investigate the gene expression changes that occur throughout aging in individual cell types, we examined nuclei extracted from the OFC. We generated snRNA-seq data from a total of 87 individuals (mean age = 54.85 years; range, 26–84 years; 32 women and 55 men; 54 individuals with a psychiatric disorder and 33 neurotypical individuals; Supplementary Tables 1 and 2 and Extended Data Fig. 1a), totaling around 800,000 nuclei. Neuropathological examination of the brain tissue confirmed the absence of macro- or microscopic changes, except for one individual, although cortical areas were unaffected. The two groups did not differ in age, sex, RNA integrity number (RIN) and postmortem interval (PMI) (Extended Data Fig. 1b). The median number of genes and counts per nucleus were 2,210 and 3,900, respectively. There was no difference in median number of genes and counts per nucleus between individuals with a psychiatric disease and neurotypical individuals (Extended Data Fig. 1b) and no correlation between age and PMI or between age and median number of genes or median number of counts (Extended Data Fig. 1c). However, we found a modest negative correlation between age and RIN (Extended Data Fig. 1c), which has previously been reported^17^. We applied Leiden clustering using highly variable genes to identify cell-type clusters (Fig. 1a–c; see Extended Data Fig. 2a–d for additional quality control). We identified 7 major cell types and 21 distinct cell types, including endothelial cells, glial cell types (oligodendrocytes, oligodendrocyte precursor cells (OPCs), microglia and two astrocyte subtypes (fibrous and protoplasmic) and subtypes of both excitatory and inhibitory neurons (Fig. 1a–d and Extended Data Fig. 3a–c). There was no difference in mean number of nuclei per cell type between individuals with a psychiatric disorder and neurotypical individuals (Supplementary Table 3).

### Cellular composition changes with age

**Extended Data Fig. 4:**
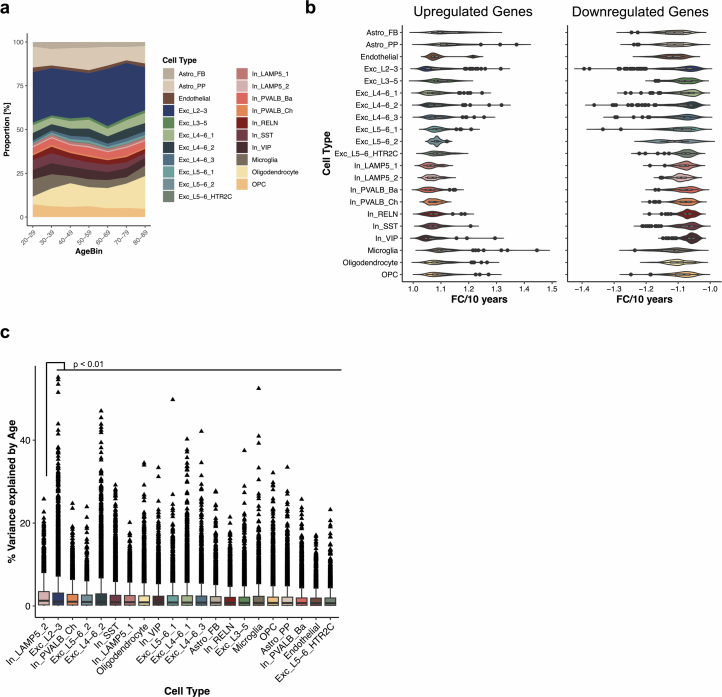
Cellular composition changes along aging, effect size distribution of differentially expressed genes, and comparison of variance in gene expression explained by age across cell types. **a**, Stacked area chart depicting cellular composition changes across age bins of 10 years. Raw proportions (uncorrected for covariates) are shown. **b**,Violin plots showing distribution of the FC per 10 years of differentially expressed genes (at FDR-adjusted p-value < 0.05) for up- (left) and downregulated (right) genes for each of the 21 cell types respectively. The number of DE genes (at FDR-adj. p-value < 0.05; N) per cell type is shown in Supplementary Table 5. Boxplot shows the median (center), IQR (bounds of box), and whiskers extending to either the maxima/minima or to the median ± 1.5× IQR, whichever is nearest. **c**, Boxplot of variance in gene expression explained by age across 21 cell types. P values were calculated by comparing variance explained by age across genes between cell types (two-sided Mann-Whitney U test) followed by multiple testing correction (fdr). For clarity only p-value for comparison between In_LAMP5_2 and all other cell types is shown and only outliers (triangles) are depicted as individual data points. Exact p-values and N (that is genes) per cell type are shown in Supplementary Table 8. Boxplot shows the median (center), IQR (bounds of box), and whiskers extending to either the maxima/minima or to the median ± 1.5× IQR, whichever is nearest.

We first investigated changes in cell composition during aging by calculating the proportions of each cell type per individual. Most cell types did not change in abundance, only the proportion of OPCs significantly decreased with age (false discovery rate (FDR)-adjusted *P* *=* 0.002), going along with a trend-line increase in oligodendrocytes (FDR-adjusted *P* *=* 0.05) and decrease in VIP inhibitory neurons (In_VIP, FDR-adjusted *P* *=* 0.05; Extended Data Fig. 4a and Supplementary Table 4).

### Aging affects the transcriptomes of all cell types

**Fig. 2:**
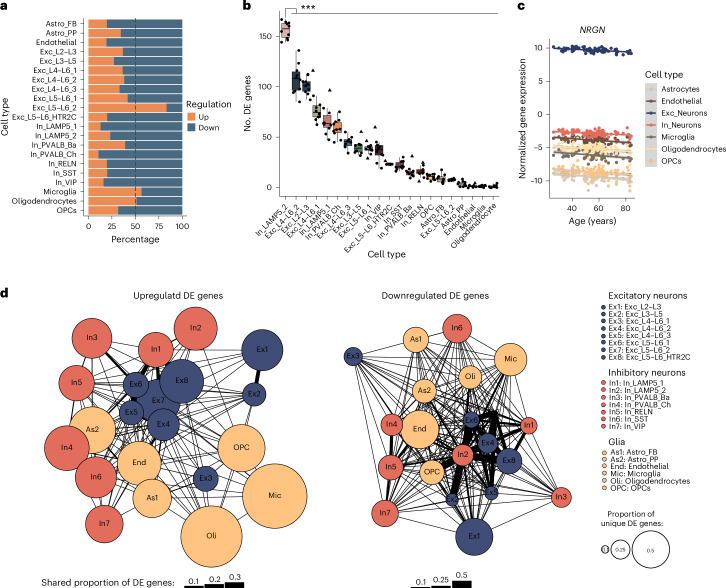
Differential gene expression analysis. **a**, Bar plot depicting the percentage of up- and downregulated DE genes (at FDR-adjusted *P* < 0.05) for the respective cell types. **b**, Box plot of the numbers of DE genes identified from the differential gene expression analysis of downsampled data (5,000 nuclei from each cell type were randomly selected ten times (that is, *N* = 10)). *P* values were calculated by comparing the numbers of DE genes between cell types (two-sided Mann–Whitney *U*-test), followed by multiple testing correction (FDR). For clarity, only the *P* value for the comparison between In_LAMP5_2 neurons and all other cell types is shown (***P < 0.001); exact *P* values are shown in Supplementary Table 7. The box plot shows the median (center) and interquartile range (IQR; bounds of the boxes), and whiskers extend to either the maxima/minima or to the median ± 1.5× IQR, whichever is nearest. Triangles indicate outliers. **c**, Scatter plot showing log normalized expression of *NRGN* corrected for covariates across aging across all major cell types. Error bands represent the 95% confidence interval. **d**, Illustration of shared and cell-type-specific DE genes for upregulated (left) and downregulated (right) DE genes (at FDR-adjusted *P* < 0.05). The number of overlapping DE genes between two cell types was normalized to the total number of DE genes of each of the two cell types, and the average was taken. The thickness of the black line between the two cell types is representative of this shared proportion of DE genes, with a thicker line indicating a higher overlap. The size of the circle for each cell type indicates the proportion of cell-type-specific DE genes, with a bigger circle indicating a higher number of unique DE genes.

**Extended Data Fig. 5:**
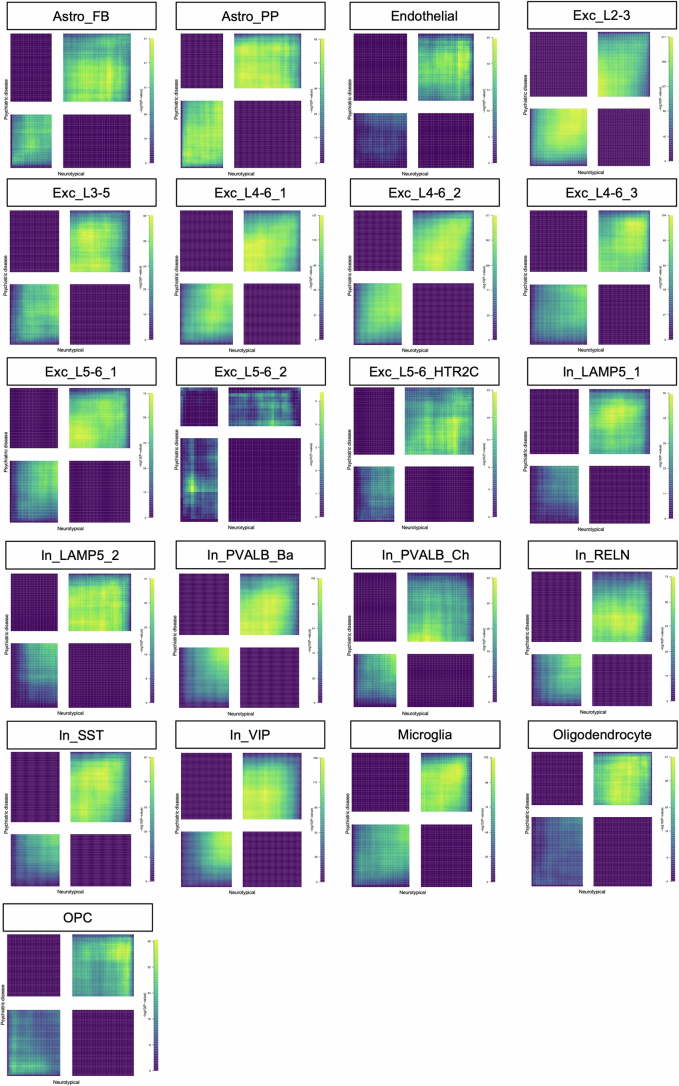
Rank-rank hypergeometric overlap of age-related gene expression pattern in controls and cases. RRHO2 plots for correspondence between differential expression results for age in neurotypical individuals (x-axis) and individuals with a pyschiatric disease (y-axis) for the respective cell type. The bottom left and top right quadrants indicate concordant overlap in genes with increased or decreased expression, respectively. In contrast, the top left and bottom right quadrants signify overlaps in genes with opposing effects between neurotypical individuals and individuals with a psychiatric disease. Genes were ranked based on the logarithm of the fold change multiplied by the negative base 10 logarithm of the uncorrected p-value from the differential expression analysis. RRHO2 employs one-sided hypergeometric tests; the color scale represents unadjusted p-values.

Using single-nucleus RNA transcriptomes, we generated pseudobulk counts for each cell type per individual to characterize cell-type-specific gene expression aging trajectories. All analyses were adjusted for covariates (disease status, sex, pH, RIN, PMI, library preparation batch and principal component 1 (PC1; for hidden confounders inferred from a batch-corrected expression matrix)) and corrected for multiple testing using the Benjamini–Hochberg (FDR) method^18^ (Methods). In total, we observed 3,299 unique differentially expressed (DE) genes with age (FDR-adjusted *P* < 0.05) across all cell types. Changes in gene expression were detected in all identified cell types, with the largest number of DE genes in upper-layer excitatory neurons (Exc_L2–L3) neurons (Supplementary Tables 5 and 6). In all cell types except oligodendrocytes, microglia and Exc_L5–L6_2 neurons, more than half of the DE genes were downregulated with increasing age (Fig. 2a), an effect previously reported in bulk brain tissue of rhesus macaques and humans^11,19^. The distribution of fold change values over a period of 10 years per cell type is shown in Extended Data Fig. 4b with great symmetry between the up- and downregulated genes in their effect size distributions. Overall, age-related gene expression direction effects were similar between neurotypical individuals and individuals with psychiatric disease across cell types as revealed by rank–rank hypergeometric overlap (RRHO; Extended Data Fig. 5). Differences in the number of DE genes among cell types are related to the statistical power to detect DE genes in a given cell type, driven by factors including the number of nuclei and sequencing reads per cell type^20^. To estimate how strongly gene expression was affected by age in each cell type, we downsampled our dataset to 5,000 nuclei per cell type, followed by differential expression analysis. This analysis showed that In_LAMP5_2 neurons, an inhibitory neuron subtype characterized by the coexpression of *LAMP5* and *LHX6* (Extended Data Fig. 3b), showed by far the most relative DE genes with age, followed by the deep-layer neuron cluster Exc_L4–L6_2 (Fig. 2b and Supplementary Table 7), a finding supported by variance partitioning analysis (Extended Data Fig. 4c and Supplementary Table 8). Interestingly, *LAMP5*^+^*LHX6*^+^ interneurons have become enriched in the cortex of primates during evolution^21^.

### Shared and unique signatures of aging across all cell types

**Fig. 3:**
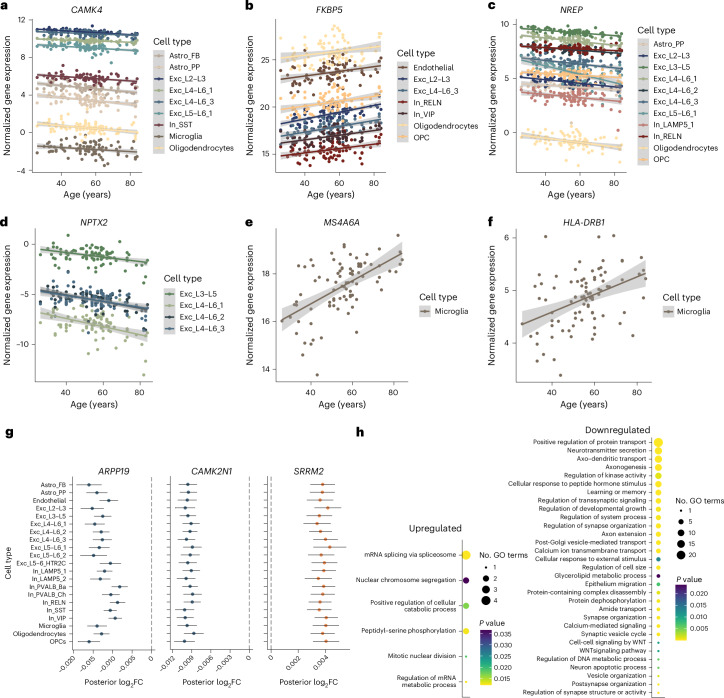
Examples of shared and cell-type-specific age-regulated genes and enriched pathways. **a**–**f**, Scatter plots showing log normalized gene expression corrected for covariates across aging of significantly DE genes in respective cell types, including *CAMK4* (**a**), *FKBP5* (**b**), *NREP* (**c**), *NPTX2* (**d**), *MS4A6A* (**e**) and *HLA-DRB1* (**f**). Error bands represent the 95% confidence interval. **g**, Forest plots showing effect sizes (posterior log_2_FC) across cell types for *ARPP19*, *CAMK2N1* and *SRRM2*. Data are represented as posterior mean ± posterior s.d.; mashR analysis was performed across all cell types (*N* = 21). **h**, Biological pathway enrichment results for up- and downregulated genes (mash analysis). Significance was determined using a one-sided Fisher’s exact test, followed by multiple testing correction (FDR). Semantic similarity analysis was used to group related GO terms. The size of each circle corresponds to the number of GO terms within the group, and the color represents the lowest *P* value among the summarized GO terms.

**Extended Data Fig. 6:**
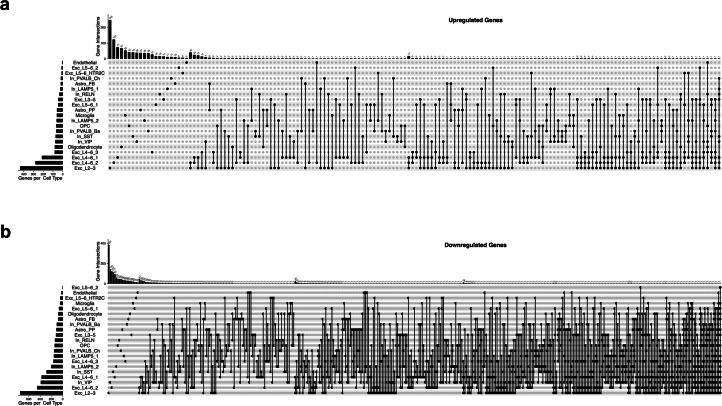
Cell-type specific and shared transcriptomic age-related genes. **a**, **b** Upset plot comparing differentially expressed genes (at FDR-adjusted p-value < 0.05) for upregulated (**a**) and downregulated (**b**) genes across 21 cell types.

Next, we compared DE genes (FDR-adjusted *P* < 0.05) across cell types. The vast majority of DE genes were unique to a single cell type, followed by shared DE genes between groups of two to three cell types (Extended Data Fig. 6a,b). *NRGN* was the only gene shared across all major cell types at this cutoff (Fig. 2c and Supplementary Table 9), whereas no common DE genes across all 21 cell types were identified (Extended Data Fig. 6a,b). Examination of the proportions of shared DE genes between cell types revealed an overall higher overlap among downregulated than among upregulated DE genes, especially across excitatory neurons (Fig. 2d). Of the DE genes at an FDR of <0.05 shared across multiple cell types, several have been previously associated with aging, such as calcium/calmodulin-dependent protein kinase IV (*CAMK4*; Fig. 3a) and FKBP prolyl isomerase 5 (*FKBP5*; Fig. 3b). *CAMK4* encodes an important transcriptional regulator previously reported to be regulated with age across several species^7^. *FKBP5* is one of the genes with the highest log_2_-transformed fold change (log_2_FC; 0.027 to 0.045 per year) value overall and with the highest upregulation with age in upper-layer excitatory neurons, as previously reported^22^. Single-nucleotide polymorphisms (SNPs) in *FKBP5* are associated with an increased risk for several psychiatric disorders, and *FKBP5* has been implicated in Alzheimer’s disease (AD) by interfering with tau processing^23,24^. Shared effects were also seen for genes relevant for neuronal differentiation and regeneration of axons—for example, *NREP* (Fig. 3c), and *NPTX2* (Fig. 3d). Microglia have a high fraction of unique DE genes (Extended Data Fig. 6a,b), including *MS4A6A*, the gene with the highest log_2_FC value of all DE genes (log_2_FC: 0.063 per year; Fig. 3e). *MS4A6A* has important roles in immunity, and SNPs within this gene are associated with AD^25,26^. *HLA-DRB1*, another unique DE gene in microglia, is significantly upregulated in expression with age and has reported genetic associations with aging (longevity^27^) and AD^28^ (Fig. 3f).

However, because statistical power influences the ability to detect significant DE genes and thus shared effects, we performed multivariate adaptive shrinkage (mash) analysis^29^ to leverage information sharing across genes and cell types. The mash analysis revealed a total of 256 shared DE genes across all 21 cell types (108 up- and 148 downregulated) at a local false sign rate of <0.05. These include, A*RPP19* (which is involved in the regulation of mitosis and is regulated with age in the brains of both humans and rhesus macaques^8,11^), *CAMK2N1* (which encodes a calcium-dependent protein kinase inhibitor with a role in synaptic long-term potentiation, a process altered during aging) and *SRRM2* (which encodes a component of the spliceosome and is implicated in neurodegenerative disorders where it mislocalizes to tau aggregates in the cytoplasm^30,31^) (Fig. 3g). This provides evidence that the large number of nuclei sequenced in our dataset allows mapping of age-related changes to individual cell types, with specific and overlapping effects, enabling insights into the cellular effects of age-related genes.

### Enrichment of biological pathways and disease

**Extended Data Fig. 7:**
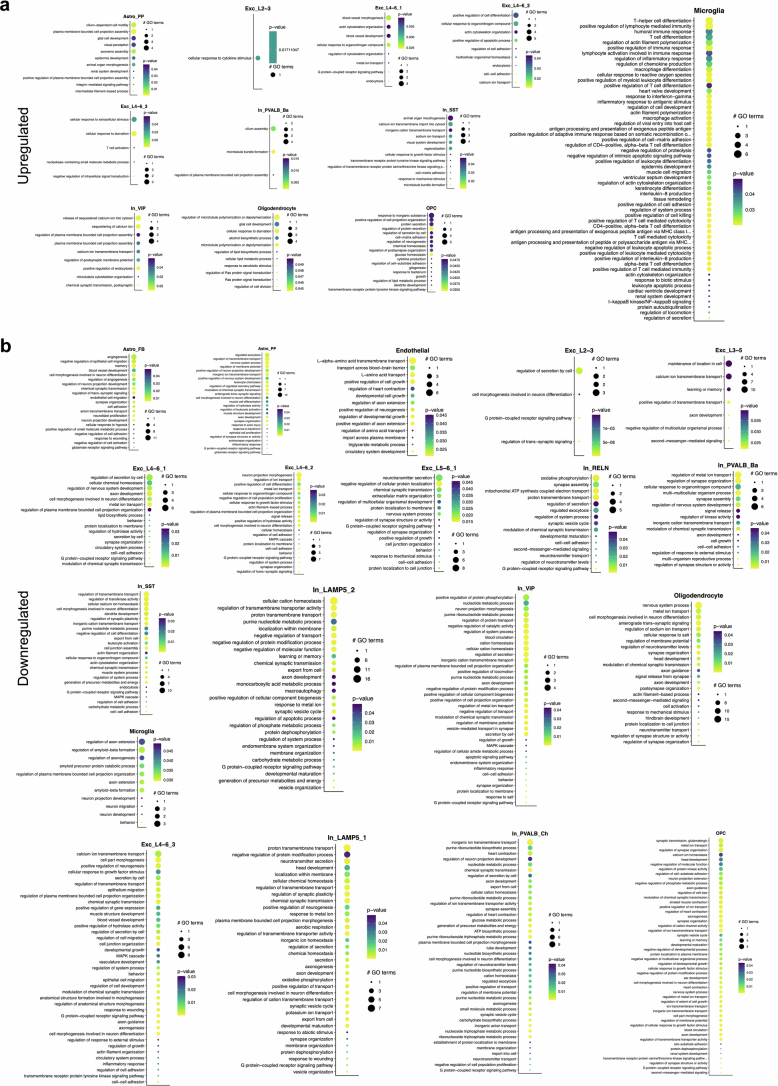
Biological pathway enrichment of age-regulated genes. **a-b**, Biological pathway enrichment results for up- (**a**) and downregulated DE genes (**b**) for each cell type. Significance was determined using the one-sided Fisher’s exact test followed by multiple testing correction (fdr). Semantic similarity analysis was employed to group related GO terms. The size of each circle corresponds to the number of GO terms within the group, and the color represents the lowest p-value among the summarized GO terms.

To better understand the shared and cell-type-specific biological processes affected by age, we performed over-representation analysis for biological pathways of the up- and downregulated genes, respectively. We started with the 256 shared genes from the mash analysis and used semantic similarity analysis to reduce redundancies in the list of significant Gene Ontology (GO) terms (Fig. 3h). Common upregulated genes are involved in processes such as mRNA splicing, which has been previously described as being affected by aging across tissues and species^32^. Downregulated genes mapped to synaptic signaling at various levels, including neurotransmitter secretion, axo-dendritic transport and (post)synapse organization, consistent with studies in human bulk brain^9,33^. Cell-type-specific biological processes (Extended Data Fig. 7) in microglia included humoral response, positive regulation of immune response and cellular response to reactive oxygen species for upregulated DE genes (Extended Data Fig. 7a), consistent with previous findings of increased immune function in the aged brain in both humans and mice^34,35^. Downregulated DE genes in microglia were enriched for terms related to the regulation of amyloid-β formation (Extended Data Fig. 7b). Within endothelial cells, downregulated DE genes showed enrichment for terms including transport across the blood–brain barrier, supporting potential disruption of the blood–brain barrier as previously shown in aged humans and mice^36,37^. Moreover, downregulation of DE genes involved in cellular ion homeostasis was observed in excitatory and inhibitory neurons. Downregulated DE genes in several inhibitory neuron subtypes mapped to metabolic processes, such as nucleotide metabolic process, and oxidative phosphorylation was seen specifically in several inhibitory neuron subtypes. In_LAMP5_2 neurons (the cell type identified as most severely affected by aging) showed enrichment for macroautophagy and regulation of apoptotic process within its downregulated DE genes. These findings show that although there are cell-type-specific pathways, there is convergence not only at the gene level but also at the pathway level (Extended Data Fig. 7).

**Fig. 4:**
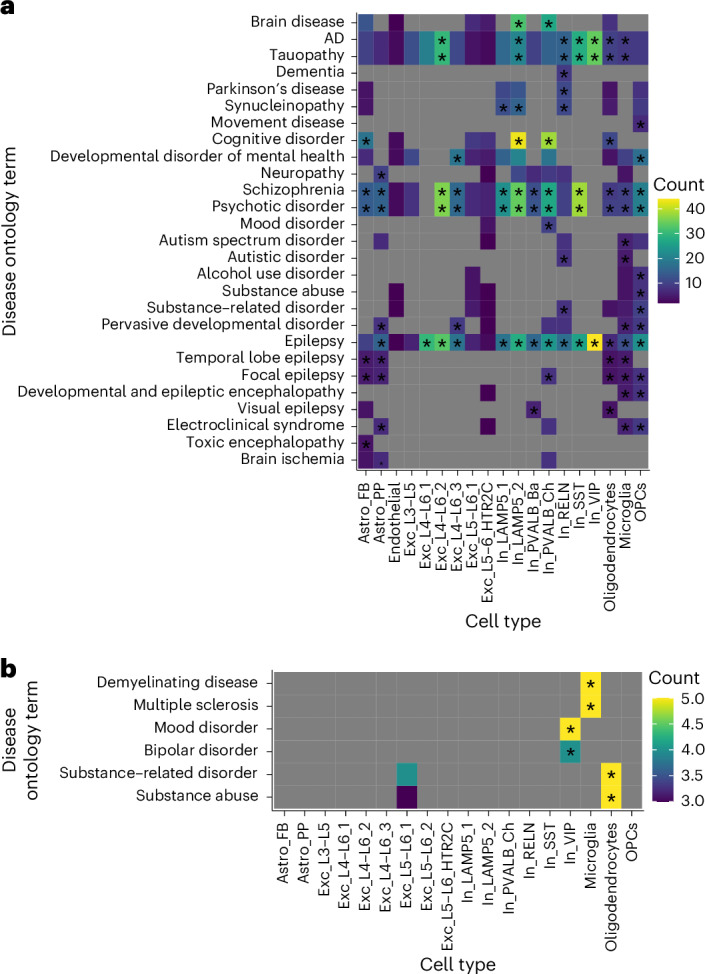
Disease enrichment for brain-related diseases of age-regulated genes. **a**,**b**, Heat maps depicting disease enrichment of age-regulated DE genes (at FDR-adjusted *P* < 0.05) across cell types for downregulated (**a**) and upregulated (**b**) DE genes. Only cell types with a minimum of one disease ontology term were included. Colors represent the number of genes (count) contributing to the disease ontology term. Significance was determined using a one-sided Fisher’s exact test, followed by multiple testing correction (FDR). Asterisks (*) indicate an FDR-adjusted *P* < 0.05. Gray values indicate not applicable. Only enrichment for brain-related diseases is shown.

**Extended Data Fig. 8:**
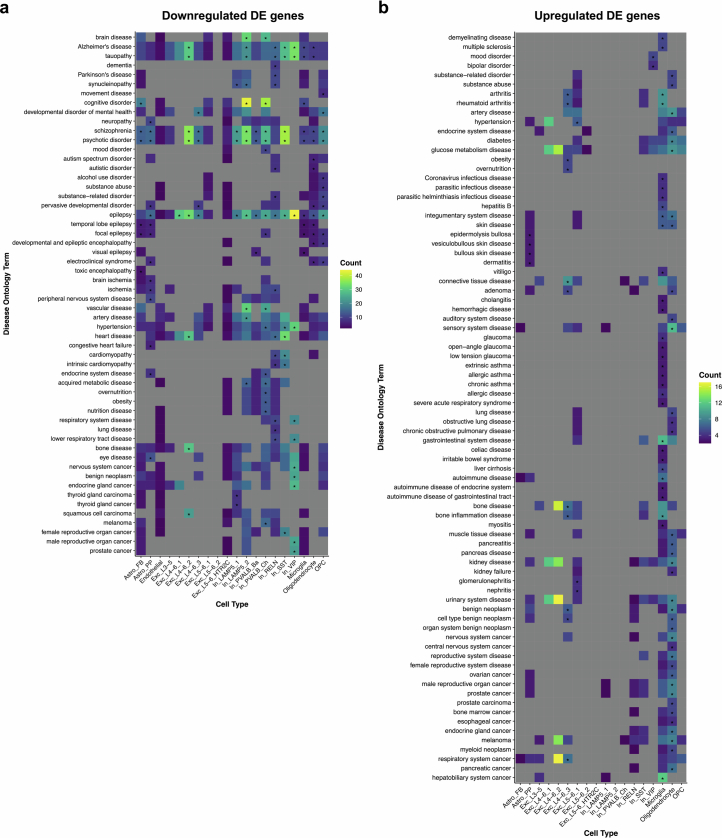
Disease enrichment of age-regulated genes. **a-b**, Heatmap depicting disease enrichment of age-regulated DE genes (at FDR-adjusted p-value < 0.05) across cell types for downregulated (**a**) and upregulated (**b**) DE genes respectively. Only cell types with minimum one disease ontology (DO) term were included. Colors represent the number of genes (count) contributing to the DO term. Significance was determined using the one-sided Fisher’s exact test followed by multiple testing correction (fdr). * asterisk indicates FDR-adjusted p-value < 0.05. Grey values indicate NA.

Disease enrichment analysis revealed that downregulated DE genes (FDR < 0.05) were enriched for genes associated with brain-related diseases, including neurodegenerative diseases (for example, AD), across various inhibitory neuron subtypes, one deep-layer excitatory neuron cell type and microglia and oligodendrocytes (Fig. 4a and Extended Data Fig. 8a). Additionally, enrichment for psychiatric disorders (for example, SCZ) was found across several excitatory, inhibitory and glial cell types. Enrichment for brain-related disorders within the upregulated DE genes included demyelinating disease (in microglia), mood disorders (in VIP inhibitory neurons) and substance abuse (in oligodendrocytes) (Fig. 4b and Extended Data Fig. 8b).

### Validation of transcriptomic changes across datasets

**Fig. 5:**
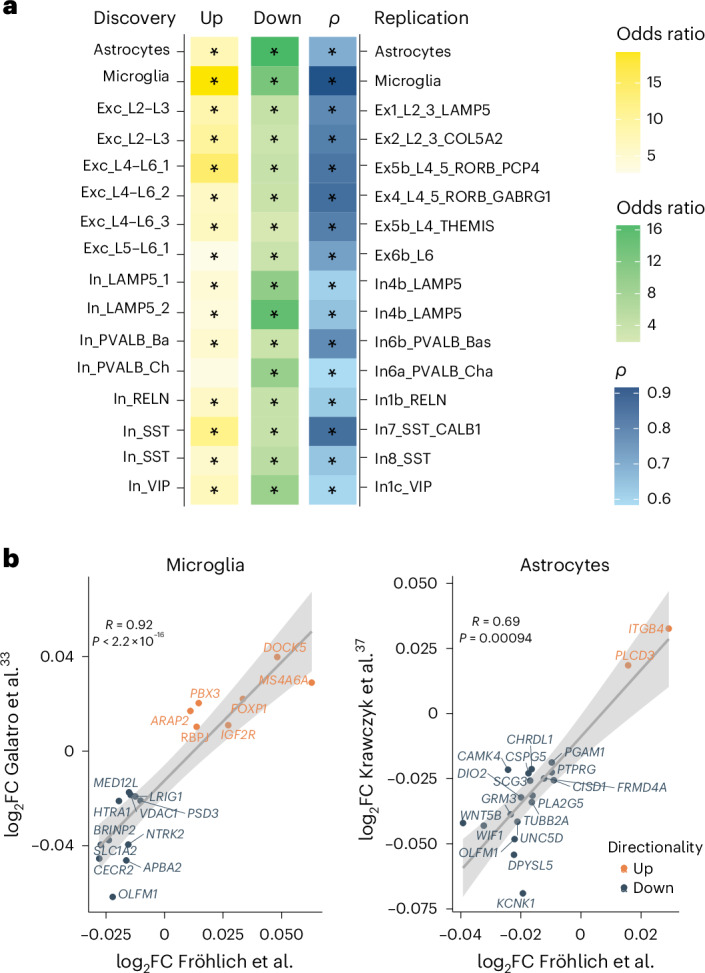
Validation of age-related genes across cell types. **a**, Heat map depicting the odds ratio of overlapping upregulated (yellow) and downregulated (green) DE genes and Spearman correlation (*ρ*; two sided) of their log_2_FC values across discovery and replication cell types. The significance of overlap was determined using a one-sided Fisher’s exact test, followed by multiple testing correction (FDR). Asterisks (*) indicate FDR-adjusted *P* < 0.05. Exact *P* values are shown in Supplementary Table 12. The replication datasets include Krawczyk et al.^38^ for astrocytes, Galatro et al.^34^ for microglia and Chatzinakos et al.^39^ for excitatory and inhibitory neurons. **b**, Scatter plots showing the log_2_FC values of overlapping DE genes in microglia (left) between this study (*x* axis) and a study by Galatro et al.^34^ (*y* axis) and the log_2_FC of overlapping DE genes in astrocytes (right) between this study (*x* axis) and a study by Krawczyk et al.^38^ (*y* axis). Genes are labeled. Orange color represents upregulated genes, whereas blue color represents downregulated genes. Significant positive correlations are indicated by Spearman’s correlation (two-sided) coefficients. Error bands represent the 95% confidence interval.

To compare our aging-related gene signature with previously published bulk datasets in the human postmortem brain, we summed all sequencing reads to a ‘full pseudobulk’ dataset and performed differential expression analysis. The identified DE genes (Supplementary Table 10) showed significant overlap with those previously reported in (pre)frontal cortex bulk data^8,9,33^ (Supplementary Table 11), emphasizing the validity of our analysis. To validate our cell-type-specific findings, we compared our identified DE genes in microglia and astrocytes (major cell-type cluster) to datasets that have identified gene expression changes over the course of aging in purified microglia^34^ and astrocytes^38^ from the cerebral cortex, respectively. Moreover, we leveraged an snRNA-seq dataset from Chatzinakos and colleagues^39^ derived from dorsolateral prefrontal cortex samples from 32 individuals with an age range of 26–60 years as a replication dataset. Within this snRNA-seq dataset, excitatory and inhibitory neuron subtypes showed sufficient power and were used for validation (see Methods for statistics). For all investigated cell types except In_PVALB_Ch neurons, Fisher’s exact test revealed a significant overlap in upregulated age-associated genes with the highest odds ratio in microglia and Exc_L4–L6_1 neurons (Fig. 5a and Supplementary Table 12). Downregulated age-associated genes significantly overlapped across all cell types, with the highest odds ratio in astrocytes, In_LAMP5_2 neurons and microglia (Fig. 5a and Supplementary Table 12). Moreover, the directionality of expression changes (log_2_FC) was highly congruent, with high correlations of the effect sizes between the overlapping DE genes (Spearman correlation coefficient (*ρ*) ranging from 0.58 in In_PVALB_Ch neurons to 0.92 in microglia) (Fig. 5a,b and Supplementary Table 12). These analyses underscore the comparability across datasets from different cohorts and cortical regions and generated using both snRNA-seq and sequencing in sorted cell populations.

### Age-associated genes enriched in genes dysregulated in AD

**Fig. 6:**
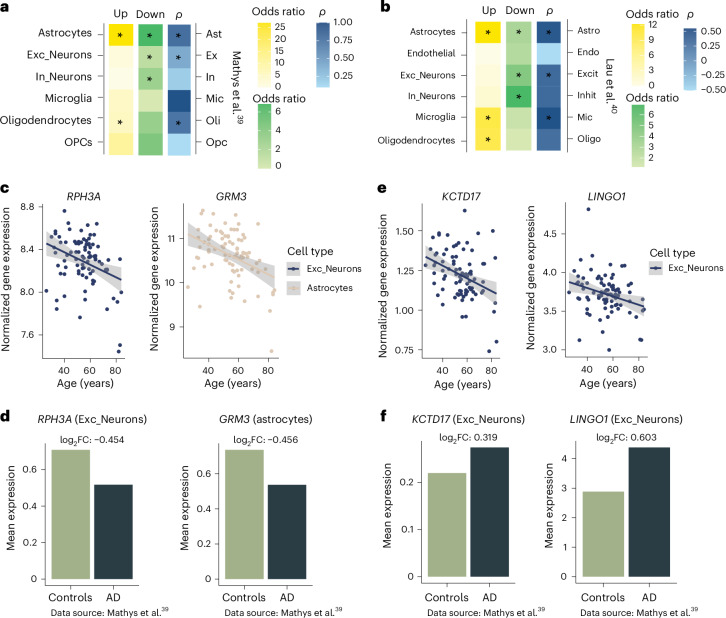
Comparison of age-regulated and AD-associated genes across cell types. **a**,**b**, Heat map depicting the odds ratio of overlapping upregulated (yellow) and downregulated (green) DE genes and Spearman correlations (*ρ*; two sided) of their log_2_FC)values for age-regulated and AD-associated genes across major cell types. The significance of overlap was determined using a one-sided Fisher’s exact test, followed by multiple testing correction (FDR). Asterisks (*) indicate FDR-adjusted *P* < 0.05. Exact *P* values are shown in Supplementary Table 13. AD datasets were from Mathys et al.^40^ (**a**) and Lau et al.^41^ (**b**). **c**,**d**, Normalized (log-transformed) gene expression corrected for covariates throughout aging of *RPH3A* and *GRM3* in respective major cell types (**c**), which show a congruent change in AD^40^. A bar plot showing the mean expression levels and log_2_FC values between neurotypical individuals and individuals with AD^40^ is also shown (**d**). **e**, Normalized (log-transformed) gene expression corrected for covariates throughout aging of *KCTD17* and *LINGO1* in excitatory neurons that show opposite directionality in AD^40^. **f**, Mean expression levels of *KCTD17* and *LINGO1* and log_2_FC values between neurotypical individuals and individuals with AD^40^ is also shown (**f**). Error bands in scatter plots represent the 95% confidence interval.

To understand the extent to which cell-type-specific DE genes associated with aging could have a role in AD, we overlapped the age-dependent DE genes with DE genes identified by snRNA-seq in the prefrontal cortex of two AD datasets^40,41^. For both datasets, we found that genes upregulated in astrocytes and oligodendrocytes in individuals with AD showed significant overlap with the age-upregulated DE genes in the corresponding major cell types in our dataset (Fig. 6a,b). Genes downregulated in excitatory and inhibitory neurons and astrocytes in individuals with AD also showed significant overlap with the age-downregulated DE genes in the corresponding cell types in our dataset (Fig. 6a,b and Supplementary Table 13). Additionally, the effect sizes (log_2_FC values) were highly correlated in astrocytes and excitatory neurons (Fig. 6a,b). Examples of genes with concordant changes with age and AD include *GRM3* in astrocytes and *RPH3A* in excitatory neurons (Fig. 6c,d). SNPs in *GRM3*, which is downregulated both with age and in AD, have been associated with increased risk for SCZ and worse cognitive function^42^. *RPH3A*, which is involved in neurotransmitter release, is downregulated in excitatory neurons with age and in AD. Higher gene expression in excitatory neurons^43^ and higher protein levels in the prefrontal cortex have been associated with cognitive resilience^44^, whereas lower protein levels have been associated with higher amyloid-β burden^45^. This supports that gradual age-related changes in these cell types could contribute to the development of AD, possibly when reaching a certain threshold level in the context of other risk factors.

Importantly, we also investigated if there are genes that are oppositely regulated between age and AD. We identified two genes with opposite cell-type-specific regulation with age versus AD that were consistent in both AD datasets. *LINGO1* and *KCTD17* decrease with age in excitatory neurons (Fig. 6e), whereas these genes are regulated in the opposite direction in AD (Fig. 6f) within the same cell type. These may represent protective factors of interest for drug targeting.

### Accelerated transcriptomic aging in psychopathology

Psychiatric disorders, transdiagnostically, are associated with lower life expectancy^46^ and an increased risk for neurodegenerative disorders^47^, which in turn is associated with an increased mortality rate^48^. Various proxies have been used to estimate biological age, such as structural magnetic resonance imaging^49^, transcriptomic data^4^ and DNA methylation (DNAm; epigenetic clocks)^50,51^. Some studies have suggested that biological aging is accelerated with psychiatric disease based on DNAm in the blood^52–54^, gene expression in the brain^4^ and magnetic resonance imaging of the brain^5^. To investigate biological age acceleration within our cohort, we calculated both epigenetic and transcriptomic age acceleration.

**Extended Data Fig. 9:**
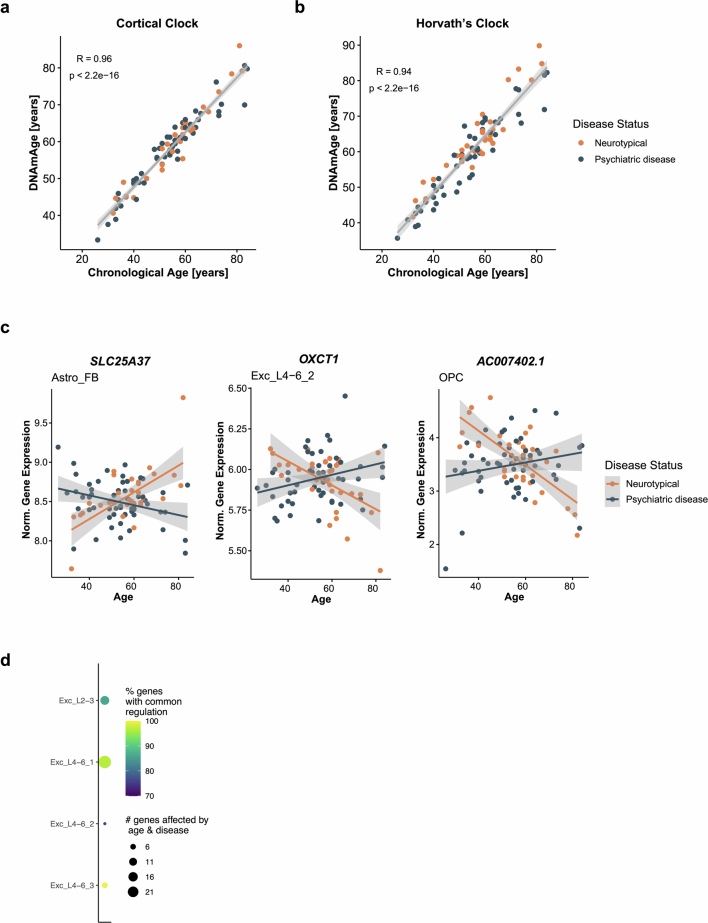
Epigenetic age, genes with interactive effects and overlap of age-regulated and disease-associated genes. **a-b**, Scatterplots showing the Pearson’s correlation (R, two-sided) between chronological age (x-axis) and DNA methylation age (DNAmAge; y-axis) as estimated using the CorticalClock (**a**) and Horvath’s multi-tissue clock (**b**). **c**, Scatterplots showing log normalized gene expression, corrected for covariates, across aging for genes showing an interactive effect between aging and disease status; *SLC25A37* in Astro_FB (left), *OXCT1* in Exc_L4-6_2 (middle), and AC007402.1 in OPC (right). Error bands represent the 95% confidence interval. **d**, Plot depicting the number of genes associated with both age and psychiatric disease. Size of the circle is proportional to the number of overlapping genes and color indicates the percentage of genes regulated in the same (common) direction across respective cell types.

We profiled bulk DNAm from the same OFC tissue using EPIC arrays and calculated DNAm age and DNAm age acceleration using Horvath’s multitissue clock^50^ and a recently developed cortical clock^51^ derived from the cortex. For both epigenetic clocks, DNAm age correlated highly with chronological age (Horvath, *r* = 0.94, *P* < 2.2 × 10^–16^; cortical clock, *r* = 0.96, *P* < 2.2 × 10^–16^; Extended Data Fig. 9a,b). However, we did not observe accelerated epigenetic aging in individuals with psychiatric diseases (Supplementary Table 14).

**Fig. 7:**
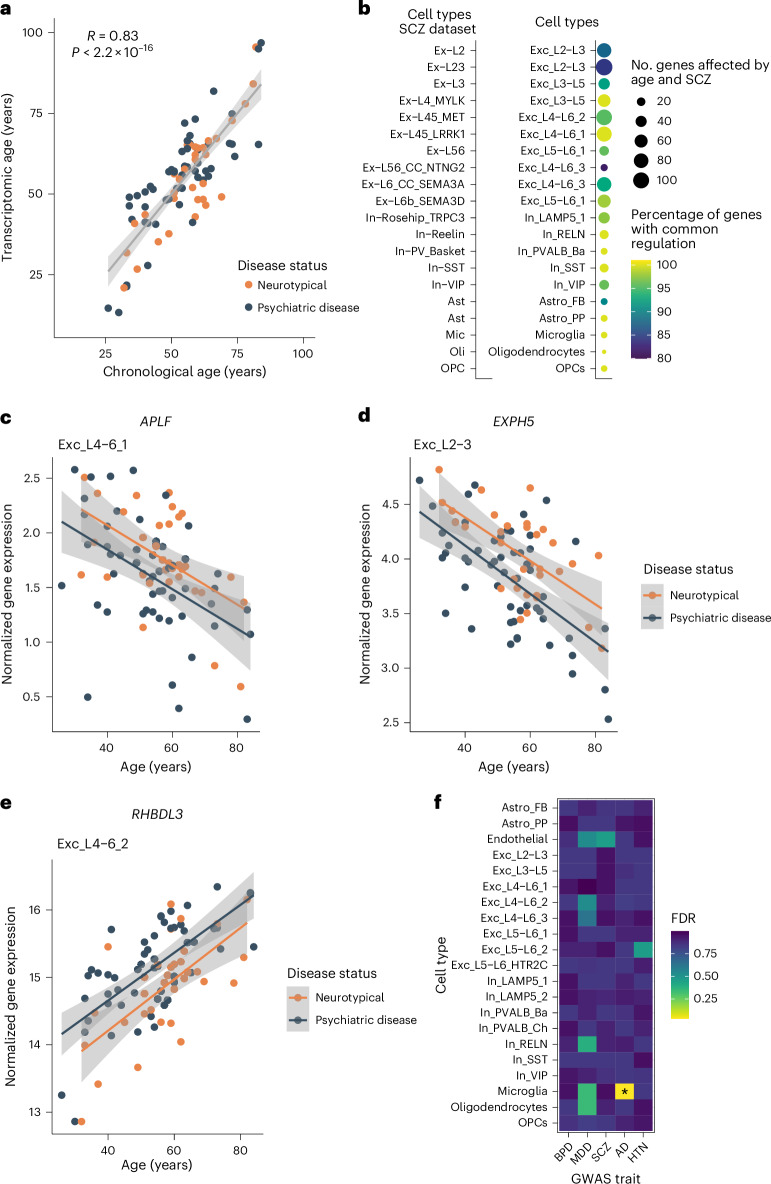
Evidence for accelerated transcriptomic age in psychopathology. **a**, Scatter plot showing the Pearson’s correlation (*R*; two sided) between chronological age (*x* axis) and transcriptomic age (transcriptomic brain age estimate; *y* axis). The error band represents the 95% confidence interval. **b**, Number of genes associated with both age and SCZ. The size of the circle is proportional to the number of overlapping genes, and color indicates the percentage of genes regulated in the same (common) direction across respective cell types. **c**–**e**, Normalized expression (log-transformed) across aging (corrected for covariates) of genes associated with both aging and disease status in respective cell types (*APLF* (**c**), *EXPH5* (**d**) and *RHBLD3* (**e**)). Error bands represent the 95% confidence interval. **f**, Heat map depicting the enrichment of genes implicated by GWAS for several traits in age-associated genes across cell types. Enrichment was tested using H-MAGMA’s two-sided gene property analysis (linear regression model), followed by multiple testing correction (FDR). Color indicates the FDR-adjusted *P* value. Asterisks (*) indicate an FDR-adjusted *P* < 0.05 (for microglia, FDR-adjusted *P* = 0.033); BPD, bipolar disorder; HTN, hypertension.

Next, we used a transcriptomic brain age predictor developed by Lin et al.^4^ to construct a transcriptomic brain age estimate and calculate transcriptomic age acceleration using our ‘full pseudobulk’ dataset. The transcriptomic brain age estimate was highly correlated with chronological age (*r* *=* 0.83, *P* < 2.2 × 10^–16^; Fig. 7a). Multiple linear regression confirmed a significant transcriptomic age acceleration in individuals with psychiatric disease compared to neurotypical individuals (*P* = 0.02; Supplementary Table 15).

Given the accelerated transcriptomic age and the disease enrichment of age-regulated genes for mental disorders reported above, we wanted to further explore how psychopathology affects aging trajectories. We therefore tested for interactive effects of age and disease status. We identified only three genes with interactive effects. These included *SLC25A37* in fibrous astrocytes, *OXCT1* in a deep-layer neuronal cluster (Exc_L4–L6_2) and *AC007402.1* in OPCs (Extended Data Fig. 9c).

We next wanted to compare age-regulated genes to genes associated with disease status. We performed differential gene expression analysis within our datasets to identify disease-associated genes (Supplementary Table 16). Disease-associated genes were identified in four excitatory neuron cell types, of which three cell types showed a significant overlap with age-regulated genes (Fisher’s exact test; FDR-adjusted *P* < 0.05; Exc_L2–L3, Exc_L4–L6_1 and Exc_L4–L6_3; Supplementary Table 17). Moreover, in all four cell types, more than 75% of overlapping genes showed concordance of expression change between age and disease (Extended Data Fig. 9d). Given that, within our dataset, we likely lacked power to detect gene expression changes associated with disease, we leveraged results from an snRNA-seq meta-analysis comparing neurotypical individuals to individuals diagnosed with SCZ^55^. Across 16 cell types, we could show that age- and SCZ-associated genes significantly overlap, and more than 80% of overlapping genes are regulated in a concordant direction (Fig. 7b and Supplementary Table 18). This supports a convergence of the signature of aging and psychopathology indicative of accelerated aging across multiple cell types. Within our dataset, genes with shifted aging trajectories in psychiatric disease include *APLF* (in Exc_L2–L3, Exc_L4–L6_1 and Exc_L4–L6_2 neurons), *EXPH5* (in Exc_L2–L3 and Exc_L4–L6_3 neurons) and *RHBDL3* (in Exc_L4–L6_2 neurons; Fig. 7c–e). *APLF* is one of the genes with the strongest decrease with age and reduction in individuals with psychiatric disease across cell types. *APLF* encodes a histone chaperone involved in DNA repair, a mechanism that has been associated with aging^56^ but so far has not been linked with psychiatric disease. *EXPH5* and *RHBDL3* have been previously associated with aging^8,33^.

**Extended Data Fig. 10:**
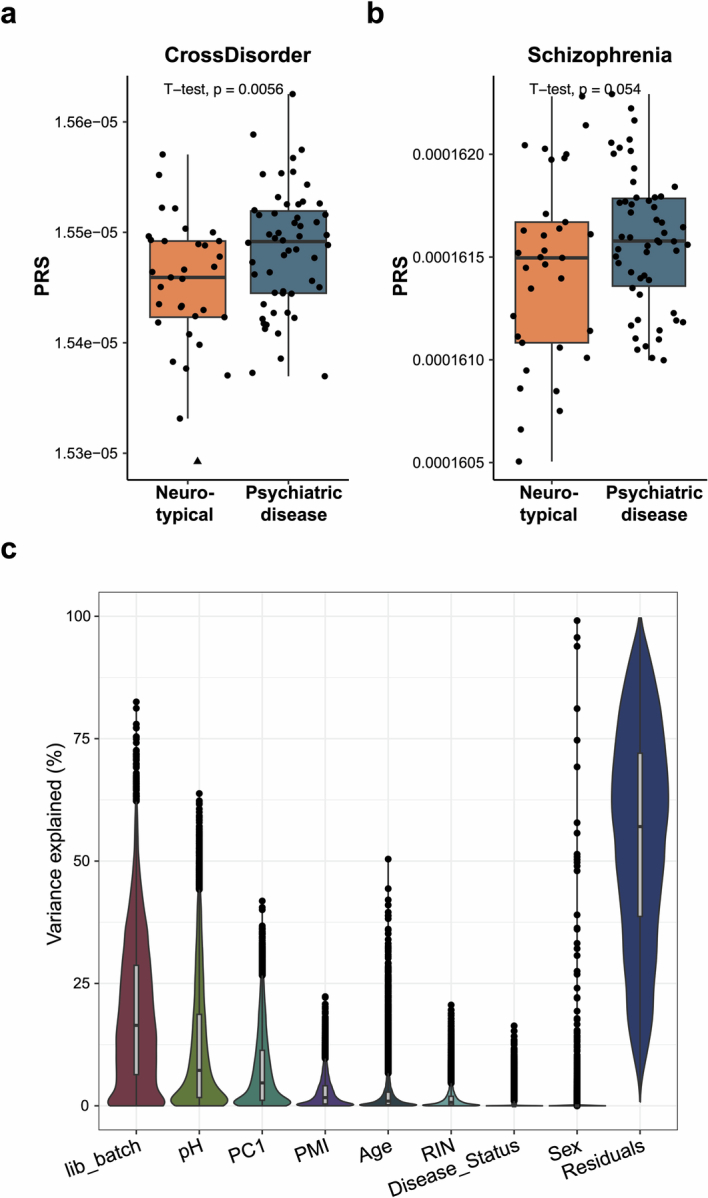
Polygenic risk for cross-disorder psychiatric phenotype and schizophrenia and variance partitioning of covariates. **a-b**, Boxplots showing polygenic risk score (PRS) for CrossDisorder (**a**) and Schizophrenia (**b**) between individuals with a pyschiatric disease (N = 54) and neurotypical individuals (N = 33). P-value of one-sided t-test is shown. Boxplot shows the median (center), IQR (bounds of box), and whiskers extending to either the maxima/minima or to the median ± 1.5× IQR, whichever is nearest. Triangles represent outliers. **c**, Violin plot of variance fractions of ‘full pseudobulk’ dataset (N = 87 biologically independent samples) for different experimental variables and covariates adjusted for in the differential expression analysis. Library preparation batch (lib_batch) explained the biggest proportion of variance in gene expression, followed by brain pH and PC1 (hidden noise). Boxplot shows the median (center), IQR (bounds of box), and whiskers extending to either the maxima/minima or to the median ± 1.5× IQR, whichever is nearest.

Risk for psychiatric disorders is conveyed by environmental and genetic factors, with notable heritability. To understand whether the convergent effects of aging and psychopathology are in part driven by genetic liability, we first calculated polygenic risk scores (PRSs) for SCZ^57^ and cross-disorder psychiatric disease^58^ (Supplementary Table 19) in our cohort. The cross-disorders PRS was significantly higher (*P* = 0.0056) in individuals with psychiatric disease, and the SCZ PRS only trend-line (*P* = 0.054), consistent with the mixed diagnosis within our cohort (Extended Data Fig. 10a). We next examined whether age-related DE genes are also identified in genome-wide association studies (GWASs) for these disorders using H-MAGMA^59^. For this, we quantified the enrichment of genes associated with several GWAS traits (bipolar disorder, major depressive disorder (MDD), SCZ, AD and hypertension (as a nonbrain-related trait)) among age-related DE genes. This analysis revealed that genes implicated by GWASs for AD are enriched in age-associated genes in microglia but not other cell types (Fig. 7f). However, we did not find enrichment for any of the other tested GWAS traits in any cell type. This suggests that age-related transcriptomic changes are not strongly influenced by genetic risk for psychiatric disorders and that the convergence of expression signatures likely reflects additional factors such as socioeconomic and behavioral changes associated with living with the disease, environmental exposures and medication.

## Discussion

In this study, snRNA-seq was performed to investigate cell-type-specific gene expression changes throughout aging in the human OFC. Our cohort comprised 87 individuals aged 26–84 years, including both neurotypical individuals and those diagnosed with a psychiatric disease (mainly SCZ), which enabled us to also investigate the effect of disease status on aging. Our study revealed that cell-type-specific gene expression changes of aging converge onto dysregulation of synaptic transmission and mRNA splicing across cell types. Notably, *LAMP5*^+^*LHX6*^+^ interneurons were identified as the cell type most strongly affected by aging. Moreover, age-associated gene expression changes across cell types were successfully replicated in independent datasets. The study also demonstrated overlapping gene expression changes between aging and AD, particularly in astrocytes and oligodendrocytes. Additionally, we observed a convergence of the transcriptomic effects of aging and psychopathology, especially SCZ, supporting findings of accelerated brain aging with psychiatric diagnoses across most cell types.

First, we examined age-related changes in cell-type proportions and found no significant changes, except for a significant decrease in OPCs, changes previously reported in animals^10,11^. However, future studies with larger sample sizes may uncover additional changes in cell-type proportions, and brain region-specific differences may exist.

Differential gene expression analysis within the identified 21 cell types indicated that all cell types are affected by aging and that the majority of age-associated transcriptional changes are cell-type specific. However, a specific cell type (inhibitory *LAMP5*^+^*LHX6*^+^ neurons (In_LAMP5_2)) seems to be most strongly affected by aging. Interestingly, this *LAMP5*^+^*LHX6*^+^ subtype has been reported to increase in abundance in the primate cortex and to most closely resemble ivy cells of the mouse hippocampus^21^. Ivy cells belong to the neuroglia-form family of cells characterized by slow spiking patterns and are involved in both feedforward and feedback inhibition^60^.

GO analysis of age-regulated genes identified disrupted synaptic signaling and mRNA splicing as converging pathways affected across cell types. This supports and extends recent findings^12^ of age-related alterations in transcriptomic latent factors enriched for genes relevant for synaptic functions across neurons and astrocytes. Several inhibitory neuron subtypes displayed dysregulation in diverse metabolic pathways and oxidative phosphorylation, indicating mitochondrial dysfunction, which has been previously described in aging and neurodegeneration^61^. Particularly, *LAMP5*^+^*LHX6*^+^ inhibitory neurons specifically exhibited dysregulation in macroautophagy and apoptosis. A recent study^62^ described reduced numbers of inhibitory LAMP5^+^ neurons in mouse models of AD and in human brains of individuals with AD, potentially driven by the here-described effects of age-associated cellular disruptions. Microglia exhibited age-related upregulation in immune pathways, consistent with previous studies of primed microglia with aging in different species^11,34,63,64^. Intriguingly, despite microglial immune activation, there was no evidence of reactive astrocytes in aging, contrasting results from Krawczyk et al.^38^ who reported an upregulation in cytokine signaling in aged astrocytes.

We validated our findings at the bulk and single-cell level. Indeed, our dataset replicated age-related changes from bulk sequencing; however, as expected, certain cell-type changes were diluted in bulk differential gene expression. We also used two studies, which had previously identified age-related genes in sorted cell populations of astrocytes^38^ and microglia^34^ by RNA-seq and another snRNA-seq dataset^39^, to replicate identified cell-type-specific DE genes and to show a significant correlation of the effect sizes (log_2_FC values) for most cell types, demonstrating comparability between methodological approaches across cohorts and cortical regions.

To relate age-associated transcriptional changes to those observed in AD, we compared our data to two independent AD snRNA-seq datasets^40,41^. This analysis revealed a convergence of age-regulated genes and genes dysregulated in AD, suggesting threshold effects contribute to disease. The most pronounced convergence occurred with upregulated genes in astrocytes and oligodendrocytes and downregulated genes in astrocytes. The overlap between age-related genes and those dysregulated in AD in astrocytes did not stem from immune-related pathways, as there was no evidence of reactive astrocytes in aging. Instead, it indicated a shared deficit in neuronal support as a common affected mechanism. Notably, two genes, *KCTD17* and *LINGO1*, exhibited opposite regulation between aging and AD in excitatory neurons. *KCTD17* encodes a member of the potassium channel tetramerization domain-containing protein family, which has been associated with neurodegeneration and psychiatric diseases^65^. *LINGO1* encodes a regulator of myelination^66^ that interacts with amyloid-β precursor protein, affecting its cleavage^67^. Interestingly, administration of anti-LINGO1 antibodies has been shown to decrease amyloid-β deposition and improve cognitive impairment in a transgenic mouse model of AD^68^. This directionality would fit to upregulation with AD and downregulation in aging not accompanied by neurodegeneration (Fig. 6e,f). *LINGO1* and *KCTD17* could thus represent interesting targets for therapeutic interventions.

Disease enrichment analyses of age-associated genes revealed enrichment of genes associated with psychiatric disorders, including SCZ, across several neuronal and glial cell types. This observation aligns with findings in bulk brain tissue of rhesus macaques^11^. Furthermore, we confirmed previously described accelerated transcriptomic age in individuals diagnosed with psychiatric disorders^4^ and identified convergent regulation of genes associated with both age and psychiatric disease using data from an snRNA-seq meta-analysis in SCZ^55^. The overlap in directionality between age- and disease-associated genes supports that aging trajectories could be shifted in psychiatric disorders, and neurodegenerating disease-relevant thresholds may be reached earlier. This could explain accelerated aging observed in individuals with psychiatric disorders and the increased risk for neurodegenerative disease in this group of individuals. Importantly, these convergent changes do not seem to be driven by genetic risk for psychiatric disease but rather reflect exposure to additional risk factors that are associated with having lived with the disease.

Certain limitations of the study should be noted. Although nuclei have been demonstrated to be comparable to whole-cell transcriptomes^69,70^, certain aspects such as mitochondrial transcription, an important pathway affected in aging and neurodegeneration, cannot be profiled. Moreover, the applied three-prime sequencing does not allow for investigation of differential splicing, another process affected in aging, neurodegeneration^71,72^ and psychiatric disorders^73,74^. Additionally, not all cells of the brain vasculature, such as pericytes or vascular smooth muscle cells, were detected, prohibiting the investigation of their transcriptomes. Although all brains were free from macro- and microscopic changes in cortical areas, contributions from pathological aging cannot be completely ruled out. The lack of evidence of accelerated epigenetic aging in psychiatric disease might be attributed to the EPIC array’s inability to capture specific age- and disease-related methylation changes. Given the high non-CpG methylation in neurons, alternative profiling methods for non-CpG methylation and epigenetic clocks may prove necessary. In addition, we were not sufficiently powered to investigate diagnosis- or sex-specific effects, both important research questions.

In summary, we provide a comprehensive dataset of cell-type-specific age-associated genes and pathways in the human OFC. We identify inhibitory *LAMP5*^+^*LHX6*^+^ neurons as transcriptionally most affected by aging. Notably, numerous gradual age-related changes overlap on the cell-type level with changes observed in AD. Additionally, we pinpoint genes with opposite regulation as potential targets for therapeutic interventions. Moreover, we also find evidence for accelerated transcriptomic aging in individuals with psychiatric disorders at the single-cell level, with a converging signature across multiple cell types. We envision that these data will provide a starting point for furthering our understanding of the aging process and development of new therapeutic targets for aging-associated pathologies.

## Methods

### Postmortem brain cohort

The study was approved by the Ethikkommission bei der LMU München (Ludwig Maximilians-Universität Munich Ethics Committee, 22-0523) and the Human Research Ethics Committees at the University of Wollongong (HE2018/351). Informed consent for brain autopsy was provided by the donors or their next of kin. No compensation was provided for donors or their next of kin. Donors were classified as neurotypical controls based on the absence of any psychiatric diagnosis, whereas individuals with psychiatric disease had been diagnosed with SCZ, schizoaffective disorder, bipolar disorder or MDD. None of the brain donors in this study were diagnosed with a neurodegenerative disorder. All included brains were neuropathologically examined, and Braak stage was determined. Only one individual (an individual with psychiatric disease) showed macro- and microscopic changes (Braak NFT stage III) but not in cortical areas. Fresh-frozen postmortem tissue of the OFC was obtained from the New South Wales Brain Tissue Resource Centre in Sydney, Australia, and was used for snRNA-seq. Only gray matter was sampled. BA11 was dissected from the third 8- to 10-mm coronal slice. The level was chosen based on visual inspection of neuroanatomical landmarks (primarily the straight and medial orbital gyri) in a slice anterior to the corpus callosum for comparability across donors. Sampling was performed using a straight edge razor blade. Supplementary Table 1 provides a summary of sample characteristics, and Supplementary Table 2 provides detailed information on all donor characteristics. Sample size was not predefined based on statistical power analysis but is comparable to (or even larger than) previous snRNA-seq studies in human postmortem brain^40,41,75,76^ and was based on tissue availability.

### Nuclei extraction

Nuclei were extracted from 50–60 mg of frozen tissue following a modified version of a published protocol^77^. In brief, nuclei were obtained using Dounce homogenization on ice in 1 ml of nucleus extraction buffer (10 mM Tris-HCl (pH 8.1), 0.1 mM EDTA, 0.32 M sucrose, 3 mM magnesium acetate, 5 mM CaCl_2_, 0.1% IGEPAL CA-630 and 40 U ml^–1^ RiboLock RNase-Inhibitor (Thermo Scientific)). Samples were layered onto 1.8 ml of sucrose cushion (10 mM Tris-HCl (pH 8.1), 1.8 M sucrose and 3 mM magnesium acetate), followed by ultracentrifugation at 107,200*g* at 4 °C for 2.5 h (Thermo Scientific Sorvall WX+ ultracentrifuge). The supernatant was discarded using vacuum suction, and nuclei were diluted in 80 µl of resuspension buffer (1× PBS, 3 mM magnesium acetate, 5 mM CaCl_2_, 1% bovine serum albumin and 40 U ml^–1^ RiboLock RNase-Inhibitor). Nuclei were filtered through a preseparation filter (20 µm; Miltenyi Biotec), stained with DAPI (1:1,000) and quantified on a hemocytometer.

### Library preparation

snRNA-seq libraries were prepared following the manufacturer’s instructions in the 10x Genomics user guide (Chromium Single Cell 3′ Reagents kit v3.1). We targeted a recovery of 10,000 nuclei per sample. Equimolar amounts of each library were pooled, subsequently treated with Illumina Free Adapter Blocking Reagent and sequenced in two batches on a NovaSeq 6000 System (Illumina).

### Sequence alignment, filtering, normalization and clustering

Sequence reads were demultiplexed using the sample index and aligned to a pre-mRNA reference, and unique molecular identifiers were counted after demultiplexing of nuclei barcodes using Cell Ranger v6.0.1 (10x Genomics). Reads were downsampled per nucleus to the 75% quartile of reads per cell (14,786 reads). Count matrices of all individuals were combined and further processed using Scanpy (v1.7.1)^78^. Nuclei were filtered according to counts, minimum genes expressed and percentage of mitochondrial genes (nuclei with <500 counts, <300 genes or a mitochondrial percentage of ≥15 were removed). Genes expressed in <500 nuclei were removed. Doublets were filtered out using DoubletDetection v3.0 (https://zenodo.org/records/6349517#.ZHdK4-xBxAc). Data were normalized using sctransform (v0.3.2)^79^. Leiden clustering using highly variable genes was applied for clustering. One cluster was removed because three individuals contributed >25% of the nuclei of that cluster, which resulted in a final dataset with 787,685 nuclei.

### Cell-type assignment

A label transfer algorithm (scarches v0.4.0 (ref. ^80^)) was used for an initial cell-type assignment. Cell-type labels from the Allen Brain Atlas (Human Multiple Cortical Areas SMART-seq, available at https://portal.brain-map.org/atlases-and-data/rnaseq/human-multiple-cortical-areas-smart-seq) were taken as a reference for our dataset. These initial assignments were refined by a manual curation based on marker gene expression^75,76,81^.

Known marker genes for major cell types included astrocytes (*AQP4*, *GFAP* and *GJA1*), endothelial cells (*CLDN5*, *FLT1* and *SYNE2*), excitatory neurons (*SLC17A7*, *SLC17A6* and *SATB2*), inhibitory neurons (*GAD1*, *GAD2* and *NXPH1*), microglia (*APBB1IP*, *C3* and *P2RY12*), oligodendrocytes (*MPB*, *MOBP* and *PLP1*) and OPCs (*OLIG1*, *PDGFRA* and *VCAN*).

Two subtypes of astrocytes were identified based on higher *GFAP* and *ARHGEF4* expression in fibrous astrocytes and higher expression of *ATP1A2, GJA1* and *SGCD* in protoplasmic astrocytes^75^. Subtypes of excitatory neurons were assigned based on the expression of cortical layer-specific marker genes (layers 2–3: *CUX2* and *RFX3*; layer 4: *IL1RAPL2*, *CRIM1* and *RORB*; layers 5–6: *RXFP1*, *TOX*, *DLC1* and *TLE4* (refs. ^75,76^)). Subtypes of inhibitory neurons were assigned based on the expression of interneuron markers *LAMP5*, *PVALB*, *RELN*, *SST* and *VIP*. For PVALB inhibitory neurons, two subtypes (basket cells (In_PVALB_Ba) and chandelier cells (In_PVALB_Ch)) could be identified (based on the high expression of *RORA*, *TRPS1*, *NFIB* and *UNC5B* in chandelier cells as described by Bakken et al.^81^). For the identification of subtypes within the In_LAMP5 cluster, Leiden clustering restricted to this cluster was performed, resulting in two subtypes (In_LAMP5_1 and In_LAMP5_2).

### Selection of covariates for differential expression analysis

Given that technical covariates are assumed to be the same across all cell types, we created a full pseudobulk count matrix by summing the gene-wise counts over all cell types and applied a stringent filter to obtain only genes of a minimum of ten counts in 90% of the individuals. Variance stabilizing transformation (vsd, DESeq2, v1.42.0)^82^ was applied, and PC analysis (PCAtools v2.14.0)^83^ was performed. Significant correlation of continuous variables with PCs was observed for RIN, PMI, pH and age. Canonical correlation analysis further identified library preparation batch (lib_batch) as a covariate. Sex and disease status (0 = control and 1 = psychiatric case) were included as covariates. To account for hidden confounders, we performed PC analysis after having calculated the normfactors, performed voom transformation and removed batch effects of all covariates using removeBatchEffect^84^ to obtain a batch-corrected expression matrix. The first PC was included as an additional covariate, resulting in the final model: (~age + disease status + sex + pH + RIN + PMI + lib_batch + PC1). Variance partitioning^85^ (variancePartition v1.33.0) was applied to obtain the variance explained by each of the covariates (Extended Data Fig. 10c). The RIN was not available for one individual and thus was set to the median.

### Differential expression analysis

We performed differential gene expression analysis using the R package dreamlet (v1.1.1)^86^, which uses a pseudobulk approach summing the gene-wise counts within the 21 identified cell types and the 7 major cell-type clusters, respectively. Additionally, we generated a ‘full pseudobulk’ count matrix by summing the gene-wise counts over all cell types for comparison with previously published bulk datasets. Normalization was performed using the processAssays function with genes and nuclei being filtered based on the following cutoffs: min.count = 10, min.prop = 0.8, min.cells = 5 and min.samples = 61. Using dreamlet’s function dreamlet, we performed differential gene expression analysis including the selected covariates. To identify age-regulated genes, we modeled library preparation batch (lib_batch), sex and disease status as random effects. *P* values were adjusted for multiple testing using the FDR method considering all tested genes across all cell types. Age-regulated DE genes with an adjusted *P* value of <0.05 were considered for downstream analysis unless otherwise specified. For easier readability, genes more highly expressed in older individuals will be referred to as ‘upregulated’, whereas genes more lowly expressed in older individuals will be referred to as ‘downregulated’.

To examine shared age-related gene expression changes across all cell types, we performed mash analysis (mashR v.0.2.79)^29^ to leverage information sharing across genes and cell types using dreamlet’s run_mash function. Genes were considered significant at a local false sign rate of <0.05.

To identify disease-associated genes, we modeled lib_batch and sex as random effects. *P* values were adjusted for multiple testing using the FDR method considering all tested genes within the respective cell type. DE genes with an adjusted *P* value of <0.1 were considered for downstream analysis.

To identify genes with an interactive effect between age and disease status, the interaction term age:disease status was included. *P* values were adjusted for multiple testing using the FDR method considering all tested genes within the respective cell type, and differences were considered significant at an FDR-adjusted *P* < 0.1.

As a similarity measure of the DE genes between two cell types (*A* and *B*), the overlap index (*O*) was calculated using the following formula and then visualized using qgraph (v1.9.8)^87^:

$$O(A,B)=(\frac{{\rm{|}}A\cap B{\rm{|}}}{{\rm{|}}A{\rm{|}}}+\frac{{\rm{|}}A\cap B{\rm{|}}}{{\rm{|}}B{\rm{|}}})/2$$

This overlap index is similar to the Jaccard index but differs, however, in the fact that the overlap proportion is considered in comparison to each of the two cell types separately and not the union (as for the Jaccard index), giving equal weight to each of the cell types (which may have large differences in the number of DE genes).

### Downsampling to determine the most strongly affected cell type by aging

To calculate the number of age-associated DE genes across cell types normalized to the number of nuclei in each cell type, 5,000 nuclei from each cell type were randomly selected ten times. Differential expression analysis was then performed for each of the ten randomly selected subsets. Next, the average number of DE genes for each cell type was calculated. To assess whether there were differences in the number of DE genes per cell type in the downsampling analyses, we compared the number of DE genes across cell types using a Mann–Whitney *U*-test. *P* values were adjusted for multiple testing using the FDR method.

### RRHO analysis to compare age-related gene expression patterns between neurotypical individuals and individuals with psychiatric disease

To compare overall gene expression patterns between neurotypical individuals and individuals with psychiatric disease, we split the snRNA-seq dataset and performed differential gene expression analysis (as described above) to identify age-regulated genes in the two groups, respectively. We next performed rank–rank hypergeometric overlap analysis using the RRHO2 package (v1.0)^88,89^ by ranking the genes according to the log_2_FC value multiplied by the negative base 10 logarithm of the uncorrected *P* value from differential expression analysis.

### Visualization of DE genes

For visualization (ggplot2 (v3.4.4)^90^ and ggpubr v0.6.0 (ref. ^91^) of DE genes (Figs. 2c, 3a–f, 6c,e and 7c–e and Extended Data Fig. 9c), cell-type-specific pseudobulk matrices (filtered for genes with a minimum of ten counts in 60% of the individuals) were normalized using the calcNormFactors function (edgeR, v4.0.1)^92^ followed by voom transformation (limma, v3.58.1)^84^. To visualize age-related genes, batch effects (disease status + sex + pH + RIN + PMI + lib_batch + PC1) were then removed using the function removeBatchEffect (limma, v3.58.1)^84^. To visualize age- and disease-related genes, batch effects (sex + pH + RIN + PMI + lib_batch + PC1) were removed using the function removeBatchEffect (limma, v3.58.1)^84^.

### Cell-type composition analysis

For each individual, we calculated cell-type proportions of each cell type by dividing the number of nuclei in a specific cell type by the total number of nuclei of the respective individual. We then used multiple linear regression to test for associations between age and cell-type composition for each cell type controlling for covariates (sex, disease status, pH, RIN, PMI and lib_batch). Associations were considered significant at an FDR-adjusted *P* < 0.05.

### Comparison of gene expression changes to previously published data

For all datasets, the significance of overlap was determined using a Fisher’s exact test (R package GeneOverlap)^93^.

#### Validation of age-regulated genes from bulk datasets

For comparison with previously identified age-related genes in bulk brain tissue (cortex), three datasets (Gonzalez-Velasco et al.^33^, Kumar et al. (frontal cortex)^8^ and Lu et al. (frontal pole)^9^) were used. DE genes from the validation datasets not tested (expressed) in the ‘full pseudobulk’ count matrix were removed. Gonzalez-Velasco et al.^33^ identified age-regulated genes in a meta-analysis across three datasets of the cortex. DE genes were split into up- and downregulated genes, respectively, and were tested for significant overlap. The DE genes in the Kumar et al.^8^ dataset were filtered for the significant genes in both the discovery and replication datasets in the frontal cortex. Gene symbols were mapped to Ensembl IDs. Because the directionality of gene expression change was not available in the supplementary data, overlap was tested independent of directionality of expression change. The DE probes identified using Affymetrix HG-U95Av2 by Lu et al.^9^ were mapped to Ensembl IDs. DE genes were split into up- and downregulated genes, respectively, and were tested for significant overlap.

#### Cell-type-specific validation of age-regulated genes

To validate our cell-type specific findings, we compared our identified DE genes (FDR-adjusted *P* < 0.05) in microglia and astrocytes (major cell-type cluster) to datasets that identified gene expression changes over the course of aging in purified microglia from the parietal cortex (Galatro et al.^34^, FDR-adjusted *P* < 0.05) and astrocytes derived from the cerebral cortex obtained during brain surgery (Krawczyk et al.^38^, FDR-adjusted *P* < 0.05), respectively. Ensembl IDs not tested (expressed) in the microglia/astrocyte (major cell-type cluster) pseudoexpression matrix were removed. Furthermore, we leveraged an snRNA-seq dataset of the dorsolateral prefrontal cortex^39^. Differential expression analysis to identify age-regulated genes was performed on the summed pseudobulk expression matrix that was filtered and voom normalized. Differential expression analysis for age was performed with limma^84^ adjusting for age, sex, PMI, genetic PC1, primary psychiatric diagnosis (that is, neurotypical, MDD and post-traumatic stress disorder), lifetime antipsychotic use, day of the experiment, percentage of cells in the cell cluster over the total number of cells and batch. Given the smaller sample size (*N* = 32) of this replication dataset and thus reduced power to detect age-regulated genes, we examined the *P* value distribution of age-regulated genes. Cell types in which the 15th percentile of nominal *P* values was <0.1 were chosen for validation, and genes with a nominal *P* value of <0.05 were considered. Of these DE genes, genes not tested (expressed) in the pseudoexpression matrix were removed. DE genes were split into up- and downregulated genes and were tested for significant overlap with our respective DE genes in the corresponding cell type. We also conducted a Spearman correlation of the fold change values. *P* values were adjusted for multiple testing using the FDR method^18^.

#### Cell-type-specific comparison of age-regulated genes to AD-associated genes

To compare DE genes associated with aging (age DE genes) to genes dysregulated in AD, we used two studies that had identified cell-type-specific DE genes in AD in the prefrontal cortex^40,41^. Both single-nucleus AD studies had only assigned major cell types (excitatory neurons, inhibitory neurons, astrocytes, endothelial cells, microglia, oligodendrocytes and OPCs (only in Mathys et al.^40^)). We used the cell-type-specific up- and downregulated AD-associated DE genes and overlapped them with the up- and downregulated age-associated DE genes (at an FDR-adjusted *P* < 0.1) of the corresponding major cell types after removing the AD-associated DE genes not expressed in the respective cell-type cluster. Moreover, we calculated a Spearman correlation of the fold change values.

#### Cell-type-specific comparison of age-associated genes with psychopathology-associated genes

To compare DE genes associated with aging (age DE genes) to genes associated with psychiatric disease, we overlapped age-associated genes and disease status-associated genes identified using our differential expression analysis as detailed above.

Moreover, to test for enrichment of DE genes associated with aging (age DE genes) with genes associated with SCZ, we leveraged results from an snRNA-seq meta-analysis comparing healthy control individuals to individuals diagnosed with SCZ^55^. We used the cell-type-specific SCZ-associated DE genes (FDR-adjusted *P* < 0.05 and absolute log_2_FC of >0.1) and overlapped them with the age-associated DE genes (at an FDR-adjusted *P* < 0.1) of the corresponding cell types after removing the SCZ-associated DE genes not expressed in the respective cell-type cluster.

### GO and disease enrichment analysis

We performed over-representation analysis of biological processes using clusterProfiler (v4.10.0)^94^ and over-representation analysis of diseases using DOSE (v3.28.0)^95^ for up- and downregulated age-associated DE genes. For shared genes across cell types (mash results), all genes expressed in at least one cell type were considered background. For cell-type-specific enrichment, all genes tested in the respective cell type were considered background. We accounted for the differences in the number of DE genes for the different cell types by only considering GO/disease terms with a minimum of 5% of DE genes overlapping with the term genes and at least two genes per term. GO terms were considered significant at an FDR-adjusted *P* < 0.05. We then used GO-Figure! (v1.0.1)^96^ to reduce the redundancy of the list of GO terms.

### DNA extraction

Genomic DNA was extracted from ~10 mg of frozen OFC tissue using a QIAamp DNA mini kit (Qiagen) following the manufacturer’s instructions (‘Protocol: DNA Purification from Tissues’) without performing the RNase A treatment. DNA samples were concentrated using a DNA Clean & Concentrator-5 kit (Zymo Research).

### DNAm measurement and calculation of epigenetic clocks

Bisulfite conversion of 400 ng of DNA was performed using an EZ-96 DNA Methylation kit (Zymo Research). Epigenome-wide DNAm analysis was performed with an Illumina Infinium MethylationEPIC BeadChip (Illumina) according to the manufacturer’s guidelines.

DNAm data were processed differently for both DNAm clocks following the original pipeline of each clock as suggested by the authors. For Horvath’s multitissue clock^50^, raw intensity values were transformed into β-values, and quality control was performed with the minfi R package (v1.36.0)^97,98^. DNAm data were then normalized with stratified quantile normalization^99^ and subsequent β-mixture quantile normalization^100^.

For the cortical DNAm clock^51^, raw intensity values were preprocessed using the watermelon (v1.34.0) and bigmelon (v1.16.0) R packages as described in detail in Shireby et al. (DNAmClockCortical preprocessing pipeline)^51,101,102^.

In both pipelines, no samples needed to be excluded due to quality control issues (mean detection *P* value of > 0.05, distribution artifacts in raw β-values or sex mismatches). In both pipelines, PC analysis was performed separately after transformation of β-values to *M* values to check for outlier samples (>3 s.d. on the two first PCs; none were excluded). We then corrected technical batch effects sequentially with ComBat within the sva R package (v3.38.0)^103^ for the strongest associations with the PCs (array and row). Batch-corrected *M* values were transformed into β-values. Further, brain tissue-related variables, which significantly correlated with the first five PCs (brain pH and storage time), were included as a covariate in all analyses.

DNAm data were used to calculate epigenetic age and epigenetic age acceleration (that is, residuals from a regression of estimated epigenetic age on chronological age adjusting for brain pH and storage time) for postmortem brain samples for the following estimators: Horvaths’ multitissue clock (with the methylclock R package^50,104^; v0.7.7) and cortical clock available code from Shireby et al.^51^ (https://github.com/gemmashireby/CorticalClock). Proportions of neuronal cells were calculated from the epigenome-wide DNAm data as suggested by Guintivano et al.^105^. Next, we used multiple linear regression to examine the association between disease status and epigenetic age acceleration, controlling for covariates (sex, smoking status and proportions of neuronal cells). Because one individual could not be run on the EPIC due to low DNA yield, and seven individuals had an unknown smoking status, the final cohort for multiple linear regression analysis consisted of 79 individuals (neurotypical individuals, *n* = 27, individuals with psychiatric disease, *n* = 52).

### Calculation of transcriptomic age

We generated a ‘full pseudobulk’ count matrix by summing the gene-wise counts over all cell types and filtered for genes with a minimum of ten counts in 60% of the individuals for the calculation of transcriptomic brain age. Counts were normalized using the calcNormFactors function (edgeR^92^), followed by voom transformation (limma, v3.58.1,^84^). Lin et al.^4^ had identified 76 genes predictive of age in the postmortem brain (BA11). Gene symbols were mapped to Ensembl IDs. Ensembl IDs not expressed in the full bulk dataset were removed. Of the 76 genes, 73 were expressed. The three missing genes were *APLNR*, *KCNA6* and *MIR29C*. To obtain the transcriptomic age estimate, the gene expression value was multiplied by its provided coefficient (weight) and summed for all 73 genes. A linear regression was fit between chronological age and transcriptomic age to rescale the unit of the transcriptomic age back to the unit of chronological age by year. To calculate age acceleration, we regressed transcriptomic age estimates on chronological age adjusting for the library preparation batch (lib_batch; the strongest batch effect). We then used multiple linear regression to examine disease status in association with transcriptomic age acceleration, controlling for covariates (sex, pH, RIN, PMI and PC1).

### SNP genotyping

Genome-wide SNP genotyping was performed on Illumina GSA-24v3-0_A1 arrays according to the manufacturer’s guidelines (Illumina). Genotypic quality control was performed using PLINK (v1.90b4.1)^106^. SNPs with a callrate of <98%, minor allele frequency of <1% or a *P* value for deviation from Hardy–Weinberg equilibrium of <1 × 10^−5^ were removed. Furthermore, individuals with a callrate of <98% were excluded. If a pair of individuals presented with a relatedness (pihat) of >0.125, the individual with the higher callrate was kept in the analysis. Individuals who were genetic outliers (more than 4 s.d. on the first three multidimensional scaling components of the identity-by-state (IBS) matrix after linkage disequilibrium (LD) pruning) were also excluded. After quality control, genotypes were subjected to imputation. Imputation was performed using shapeit2 (v2.r837)^107^ and impute2 (v2.3.2)^108^ using the 1000 Genomes Phase III reference sample. After imputation, SNPs with an info score below 0.6, with a minor allele frequency below 1% or that deviated from Hardy–Weinberg equilibrium (*P* < 1 × 10^−5^) were excluded from further analysis, resulting in 9,652,209 SNPs.

### Calculation of PRSs

PRSs were calculated based on GWASs for a cross-disorder phenotype^58^ and SCZ^57^.The PRS-CS (v1.0.0) package^109^ was applied in Python (v3.6.8) for the inference of posterior effect sizes of SNPs in the GWAS summary statistics. The linkage disequilibrium reference panel was set to the one constructed using the 1000 Genomes Project phase 3 European samples, which is also linked on the PRS-CS GitHub page (https://github.com/getian107/PRScs). Phi, the global shrinkage parameter of PRS-CS, was set to 1 × 10^–2^ for SCZ, the recommended setting for highly polygenic traits. For cross-disorder, no phi parameter was specified, as the sample size of the GWASs is sufficient to learn phi from the data. PLINK^110^ (v2.00a2.3LM, https://www.cog-genomics.org/plink/1.9/) was applied with the score parameter to calculate the PRS per sample based on the posterior effect sizes previously inferred.

### GWAS enrichment analysis

A GWAS enrichment analysis was conducted with H-MAGMA (v1.10)^59^. Significant GWAS hits were mapped to genes based on GWAS summary statistics for AD^111^, SCZ^57^, bipolar disorder^112^, MDD^113^ and hypertension (http://www.nealelab.is/blog/2017/7/19/rapid-gwas-of-thousands-of-phenotypes-for-337000-samples-in-the-uk-biobank) and the European 1,000 genomes reference panel (available at https://github.com/thewonlab/H-MAGMA). A gene-level analysis in the form of a gene property analysis was performed on the mapped results with the ‘–gene-covar’ argument in MAGMA. With this approach, the gene-level regression framework was used to examine if differential expression related to age is associated with GWAS results. Here, differential expression results were entered as a continuous variable, represented as –log_10_(*P* value) × log_2_FC.

### Reporting summary

Further information on research design is available in the Nature Portfolio Reporting Summary linked to this article.

## Online content

Any methods, additional references, Nature Portfolio reporting summaries, source data, extended data, supplementary information, acknowledgements, peer review information; details of author contributions and competing interests; and statements of data and code availability are available at 10.1038/s41593-024-01742-z.

## Supplementary information

Reporting Summary

Supplementary Table Supplementary Tables 1–19.

## Data Availability

DNAm data (EPIC arrays) have been deposited into the Gene Expression Omnibus (GEO) database under accession number GSE254293. snRNA-seq data (raw data and anndata object) have been deposited into the GEO database under the accession number GSE254569. For cell-type assignments of the snRNA-seq data, cell-type labels from the Allen Brain Atlas (Human Multiple Cortical Areas SMART-seq, available at https://portal.brain-map.org/atlases-and-data/rnaseq/human-multiple-cortical-areas-smart-seq) were taken as a reference for our dataset. The snRNA-seq replication dataset from Chatzinakos et al.^39^ is available at https://www.synapse.org/Synapse:syn33235943 (raw data) and https://www.synapse.org/Synapse:syn39718968 (metadata). For PRS calculation, GWAS summary statistics for SCZ^57^ (available at https://figshare.com/articles/dataset/scz2022/19426775) and a psychiatric cross-disorder phenotype^58^ (available at https://figshare.com/articles/dataset/cdg2019/14672034) were used. For the GWAS enrichment analysis using H-MAGMA, significant GWAS hits were mapped to genes based on GWAS summary statistics for AD^111^ (available at https://vu.data.surfsara.nl/index.php/s/l7aiRr1UEgdoJfZ), SCZ^57^ (available at https://figshare.com/articles/dataset/scz2022/19426775), bipolar disorder^112^ (available at https://figshare.com/articles/dataset/PGC3_bipolar_disorder_GWAS_summary_statistics/14102594), MDD^113^ (available at https://datashare.ed.ac.uk/handle/10283/3203) and hypertension (http://www.nealelab.is/uk-biobank, ‘GWAS round 2 results can be found here’; available at https://broad-ukb-sumstats-us-east-1.s3.amazonaws.com/round2/additive-tsvs/I9_HYPERTENSION.gwas.imputed_v3.both_sexes.tsv.bgz) and the European 1,000 genomes reference panel (available at https://github.com/thewonlab/H-MAGMA).

## Code availability

All analysis scripts are accessible in the following GitHub repository: https://github.com/AnnaSophieFroehlich/single_cell_aging.
