# Supplementary material for: Single-nucleus transcriptomic profiling of human orbitofrontal cortex reveals convergent effects of aging and psychiatric disease
Source: Nat Neurosci. 2024 Sep 3;27(10):2021–32. doi: 10.1038/s41593-024-01742-z (PMC11452345; doi:10.1038/s41593-024-01742-z)
Supplement: Supplementary file 1 — Reporting Summary [file 41593_2024_1742_MOESM1_ESM.pdf]

nature portfolio

Corresponding author(s): Anna S. Fröhlich, Elisabeth B. Binder

Last updated by author(s): Jul 16, 2024

Reporting Summary

Nature Portfolio wishes to improve the reproducibility of the work that we publish. This form provides structure for consistency and transparency in reporting. For further information on Nature Portfolio policies, see our Editorial Policies and the Editorial Policy Checklist.

Statistics

For all statistical analyses, confirm that the following items are present in the figure legend, table legend, main text, or Methods section.

n/a	Confirmed	
☐	☒	The exact sample size (*n*) for each experimental group/condition, given as a discrete number and unit of measurement
☒	☐	A statement on whether measurements were taken from distinct samples or whether the same sample was measured repeatedly
☐	☒	The statistical test(s) used AND whether they are one- or two-sided<br>*Only common tests should be described solely by name; describe more complex techniques in the Methods section.*
☐	☒	A description of all covariates tested
☐	☒	A description of any assumptions or corrections, such as tests of normality and adjustment for multiple comparisons
☐	☒	A full description of the statistical parameters including central tendency (e.g. means) or other basic estimates (e.g. regression coefficient) AND variation (e.g. standard deviation) or associated estimates of uncertainty (e.g. confidence intervals)
☐	☒	For null hypothesis testing, the test statistic (e.g. *F*, *t*, *r*) with confidence intervals, effect sizes, degrees of freedom and *P* value noted<br>*Give P values as exact values whenever suitable.*
☒	☐	For Bayesian analysis, information on the choice of priors and Markov chain Monte Carlo settings
☒	☐	For hierarchical and complex designs, identification of the appropriate level for tests and full reporting of outcomes
☐	☒	Estimates of effect sizes (e.g. Cohen's *d*, Pearson's *r*), indicating how they were calculated

Our web collection on statistics for biologists contains articles on many of the points above.

Software and code

Policy information about availability of computer code

Data collection	Next-generation sequencing data were all generated on Illumina platforms, and demultiplexed at the sequencing facility.
Data analysis	Sequencing alignment of snRNA-seq data was performed using CellRanger v6.0.1.<br>Pre-processing, filtering, normalization was performed in python v3.6.8 using scanpy v1.7.1, DoubletDetection v3.0, normalization using sctransform v0.3.2, label transfer algorithm using scarches v0.4.0.<br>After cell type assignment, summed pseudobulk count per cell type were calculated and all further analyses were conducted in R v4.3.1, DESeq2 (v1.42.0), PCAtools (v2.14.0), variancePartition (v1.33.0), dreamlet (v1.1.1), mashR (v.0.2.79), qgraph (v1.9.8), RRHO2 (v1.0) , edgeR (v4.0.1) , limma (v3.58.1), ggplot2 (v3.4.4), ggpubr (v0.6.0), GeneOverlap (v1.38.0), clusterProfiler (v4.10.0), DOSE (v3.28.0)<br>GO-Figure! (v1.0.1)<br>Preprocessing and normalization of DNA methylation data: minfi v1.36.0, watermelon v1.34.0, bigmelon (v1.16.0), sva v3.38.0, methylclock v0.7.7<br>For genotyping analysis: Plink(v1.90b4.1, shapeit2 (v2.r837), IMPUTE2 (v2.3.2)<br>For PRS calculation: PLINK v2.00a2.3LM, PRS-CS v1.0.0<br>For GWAS enrichment analysis: H-MAGMA v1.10<br>All analysis scripts are accessible in the following github repository: https://github.com/AnnaSophieFroehlich/single_cell_aging

For manuscripts utilizing custom algorithms or software that are central to the research but not yet described in published literature, software must be made available to editors and reviewers. We strongly encourage code deposition in a community repository (e.g. GitHub). See the Nature Portfolio guidelines for submitting code & software for further information.

Data

Policy information about availability of data

All manuscripts must include a data availability statement. This statement should provide the following information, where applicable:

- Accession codes, unique identifiers, or web links for publicly available datasets
- A description of any restrictions on data availability
- For clinical datasets or third party data, please ensure that the statement adheres to our policy

DNA methylation data (EPIC arrays) have been deposited into the Gene Expression Omnibus (GEO) database under accession number GSE254293; snRNA-seq data (raw data as well as anndata object) have been deposited into the GEO database under the accession number GSE254569. For cell type assignment of snRNA-seq data cell type labels from the Allen Brain Atlas (Human Multiple Cortical Areas SMART-seq, available at: https://portal.brain-map.org/atlases-and-data/rnaseq/human-multiple-cortical-areas-smart-seq) were taken as a reference for our dataset. The snRNA-seq replication dataset from Chatzinakos, C., et al. Am J Psychiatry 2023 (https://doi.org/10.1176/appi.ajp.20220478) is available at https://www.synapse.org/Synapse:syn33235943 (raw data) and at https://www.synapse.org/Synapse:syn39718968 (meta data). For PRS calculation, GWAS summary statistics for schizophrenia (10.1038/s41586-022-04434-5; available at https://figshare.com/articles/dataset/scz2022/19426775) and a psychiatric cross-disorder phenotype (10.1016/j.cell.2019.11.020; available at https://figshare.com/articles/dataset/cdg2019/14672034) were used. For the GWAS enrichment analysis using H-MAGMA, significant GWAS hits were mapped to genes based on GWAS summary statistics for Alzheimer's disease (10.1038/s41588-018-0311-9; available at https://vu.data.surfsara.nl/index.php/s/l7aiRr1UEgdoJfZ), schizophrenia (10.1038/s41586-022-04434-5; available at https://figshare.com/articles/dataset/scz2022/19426775), bipolar disorder (10.1038/s41588-021-00857-4; available at https://figshare.com/articles/dataset/PGC3_bipolar_disorder_GWAS_summary_statistics/14102594), MDD (10.1038/s41593-018-0326-7; available at https://datashare.ed.ac.uk/handle/10283/3203) and hypertension (http://www.nealelab.is/uk-biobank, "GWAS round 2 results can be found here"; available at https://broad-ukb-sumstats-us-east-1.s3.amazonaws.com/round2/additive-tsvs/I9_HYPERTENSION.gwas.imputed_v3.both_sexes.tsv.bgz) and the European 1,000 genomes reference panel (available at https://github.com/thewonlab/H-MAGMA).

Research involving human participants, their data, or biological material

Policy information about studies with human participants or human data. See also policy information about sex, gender (identity/presentation), and sexual orientation and race, ethnicity and racism.

Reporting on sex and gender	While the information from the NSW brain bank likely also included information on reported gender, we used biological sex in our analyses, as assessed from genotype information. Out of the 87 post-mortem brain samples 32 were female and 55 were male.
Reporting on race, ethnicity, or other socially relevant groupings	The 87 post-mortem brain samples were derived from individuals with European ancestry confirmed by whole-genome genotyping analysis. The choice of a 'homogenous sample' of individuals from European ancestry is based on the fact that gene expression has been shown to be influenced by the genetic make-up (ethnic background) and this was the ethnic group in the brain bank with the most samples.
Population characteristics	The 87 post-mortem brain samples were derived from a cohort of individuals between 26 and 84 years of age. 32 were female and 55 were male (biological sex). 33 were grouped as healthy controls based on the absence of a psychiatric diagnosis, whereas 54 formed part of the psychiatric cases (detailed diagnosis: N=5 with bipolar disorder, N=6 with major depression, N= 36 with schizophrenia, and N=7 with schizoaffective disorder). Supplementary Table 1 provides a summary of sample characteristics and Supplementary Table 2 provides detailed information on all donor characteristics.
Recruitment	The total number of human post-mortem tissue samples obtained from NSW Brain Tissue Resource Centre was based on tissue availability maximizing for individuals with psychiatric disorders and matched controls.
Ethics oversight	The study was approved by the Ethikkommission bei der LMU München (Ludwig Maximilians-Universität Munich Ethics Committee; 22-0523) and the Human Research Ethics Committees at the University of Wollongong (HE2018/351). Informed consent for brain autopsy was provided by the donors or their next of kin. No compensation was provided for donors or their next of kin.

Note that full information on the approval of the study protocol must also be provided in the manuscript.

Field-specific reporting

Please select the one below that is the best fit for your research. If you are not sure, read the appropriate sections before making your selection.

☒ Life sciences ☐ Behavioural & social sciences ☐ Ecological, evolutionary & environmental sciences

For a reference copy of the document with all sections, see nature.com/documents/nr-reporting-summary-flat.pdf

Life sciences study design

All studies must disclose on these points even when the disclosure is negative.

Sample size	Sample size was not pre-defined based on statistical power analysis but is comparable to (or even larger than) previous snRNA-seq studies in human postmortem brain and was based on tissue availability.
Data exclusions	Originally, 92 post-mortem brain samples were used for this study, 4 samples were excluded however due to a clog during snRNAseq library

Data exclusions	preparation and one sample was excluded due to bad data quality (too low RIN).<br><br>Lowly expressed genes were excluded from the analysis: Within the dreamlet pipeline the function processAssays() was used for normalization and included the following filter settings: min.count=10, min.prop=0.8, min.cells=5.
Replication	Using previously published data sets we replicated our findings: We used the results (age-associated genes) of 3 published gene expression datasets derived from bulk human brain tissue (Gonzalez-Velasco et al. 10.1016/j.bbagrm.2020.194491, Kumar et al. 10.1016/j.neurobiolaging.2012.10.021, and Lu et al. 10.1038/nature02661) to show significant overlap of age-associated genes with the results (age-associated genes) derived from our 'full pseudobulk' dataset. To validate our cell-type specific findings, we compared our identified age-associated DE genes in microglia and astrocytes (major cell type cluster) with data sets having identified gene expression changes over the course of aging in purified microglia from the parietal cortex (Galatro et al. 10.1038/nn.4597) and astrocytes derived from the cerebral cortex obtained during brain surgery (Krawczyk et al. https://doi.org/10.1523/JNEUROSCI.0407-21.2021) respectively and showed significant overlap in age-associated genes. Moreover, we used inhibitory and excitatory neuron clusters from a snRNA-seq data set from the dorsolateral prefrontal cortex (Chatzinakos et al. 10.1176/appi.ajp.20220478) to identify age-associated genes and showed significant overlap with age-associated genes in excitatory and inhibtory clusters identified in our snRNA-seq dataset.
Randomization	For a balanced experimental design not confounded by our variables of interest, batches for snRNA seq library prep and Illumina Infinium MethylationEPIC BeadChips were assigned using the r package OSAT (Yan et al. 2012, https://doi.org/10.1186/1471-2164-13-689).
Blinding	Investigators were not fully blinded to group allocation during data collection and analysis. Yet, samples had been assigned into balanced batches using the r package OSAT. During experimental procedures, samples were labeled only with 3-digit subject identifier, which did not reflect any information on age, sex or group allocation that could bias results.

Reporting for specific materials, systems and methods

We require information from authors about some types of materials, experimental systems and methods used in many studies. Here, indicate whether each material, system or method listed is relevant to your study. If you are not sure if a list item applies to your research, read the appropriate section before selecting a response.

Materials & experimental systems

n/a	Involved in the study
☒	☐ Antibodies
☒	☐ Eukaryotic cell lines
☒	☐ Palaeontology and archaeology
☒	☐ Animals and other organisms
☒	☐ Clinical data
☒	☐ Dual use research of concern
☒	☐ Plants

Methods

n/a	Involved in the study
☒	☐ ChIP-seq
☒	☐ Flow cytometry
☒	☐ MRI-based neuroimaging
